# Tailored Lignin Xerogels: Insights into Morphology Control

**DOI:** 10.1021/acssuschemeng.5c11885

**Published:** 2026-03-19

**Authors:** Aymane El Bouhali, Frédéric Addiego, Hande Barkan-Öztürk, Alexander Bismarck, Jean-Sébastien Thomann, Daniel F. Schmidt

**Affiliations:** † Department of Materials Research and Technology, 87145 Luxembourg Institute of Science and Technology, Esch-Sur-Alzette L-4362, Luxembourg; ‡ Department of Physics and Materials Science, University of Luxembourg, Esch-sur-Alzette L-4365, Luxembourg; § Institute of Material Chemistry and Research, Faculty of Chemistry, 27258 University of Vienna, Währing Strasse 42, Vienna 1090, Austria; ∥ Department of Chemical Engineering, Imperial College London, South Kensington Campus, London SW7 2AZ, UK

**Keywords:** lignin, sol−gel, phase separation, network formation, porous

## Abstract

Lignin, a renewable biopolymer, presents significant potential for sustainable materials development, particularly in the synthesis of porous adsorbents for water treatment. This study introduces a tailored approach for synthesizing lignin-based xerogels (LBX) via a sol–gel process combined with polymerization-induced phase separation (PIPS), enabling a controlled pore morphology and hierarchy. Data on lignin structure and molecular weight are used to effectively predict the outcome of the sol–gel process prior to the incorporation of polyethylene glycol (PEG) as an additive polymer. By systematically varying the molecular weight and concentration of PEG, the influence of these factors on phase separation dynamics, drying behavior, and the structure of the resulting porous bodies is revealed. The synthesized xerogels exhibited tunable pore structures, with average pore sizes ranging from 10 to 90 μm, porosities between 19 and 73 vol %, specific surface areas (SSAs) from 0.7 to 13.2 m^2^/g, and permeability values spanning 1.3 to 5.6 darcys. This study highlights a tunable strategy for lignin valorization, offering insights into the development of biobased porous materials with potential relevance to heavy metal adsorption.

## Graphical abstract

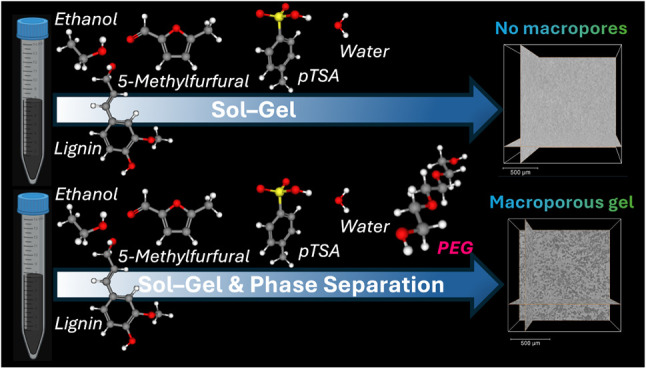

## 1 Introduction

Heavy metals such as arsenic, cadmium, chromium, mercury, lead, etc. are toxic, and can easily accumulate in the environment,^1,2^ posing significant risks to ecosystems^3^ and human health.^4^ Recently, aligning with the global shift toward sustainable chemistry, there has been a marked rise in interest surrounding biobased porous materials as effective agents for adsorbing diverse wastewater heavy metals due to their abundant reserves, low cost, and sustainable nature.^5^ Notably, lignin, a biopolymer derived from plant biomass generated as a byproduct of the pulp industry, has emerged as a prominent material with promising adsorption capabilities.^6–10^

As the second most abundant natural organic polymer compound after cellulose, lignin stands out as a unique nonpetroleum resource providing renewable aromatic compounds. Composed of three primary monolignols, *p*-coumaryl alcohol (H unit), coniferyl alcohol (G unit), and sinapyl alcohol (S unit) (Figure 1a),^11^ lignin is a highly branched polymer formed through radical coupling reactions, leading to a complex network linked by C–O and C–C bonds including the β–O–4, β–β, β–5, 5–5, and 4–O–5 interunit linkages (Figure 1b).^12^ Beyond the traditional monolignols, recent research has revealed the incorporation of additional components such as flavonoids, hydroxystilbenes, and hydroxycinnamic amides.^13^

**Figure 1:**
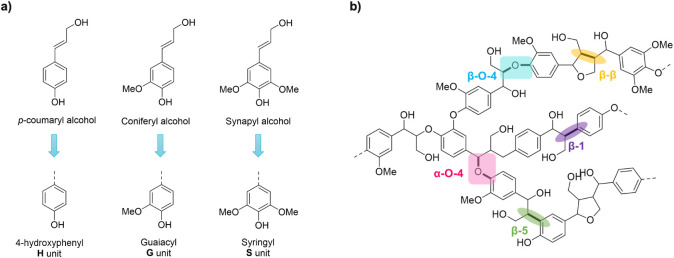
(a) Lignin monolignols and their corresponding units; (b) lignin model structure illustrating common interunit linkages.

Technical lignins refer to lignins that are obtained as byproducts of industrial processes from the delignification of lignocellulosic biomass. These lignins are called “technical” to distinguish them from “native” lignin found in natural sources such as wood and plants. They have been subjected to various extraction and processing methods, leading to differences in their molecular weight, chemical composition, and properties. Common industrial extractions of lignin include kraft, sulfite, soda, and organosolv pulping.^14^

Kraft and soda pulping are two alkaline processes. Kraft pulping employs a combination of sodium hydroxide and sodium sulfide, whereas soda pulping relies solely on sodium hydroxide for its delignification effects. Sulfite pulping is another sulfur-based separation process, but unlike kraft pulping, sulfurous acid is used in the presence of calcium or magnesium ions. In contrast, organosolv pulping uses a mixture of water and an organic solvent, often assisted by acid catalysts. This approach results in the extraction of pure lignin with lower amounts of both residual carbohydrates and ash. Carboxyls and aliphatic and phenolic hydroxyls naturally present in lignin, along with other functional groups introduced during the pulping process (e.g., thiols), provide technical lignins with chemical reactivity and modulate their properties.

While technical lignins inherently possess some limited porosity and surface area, their adsorption capacity for heavy metal ions is generally reported to be relatively low.^15,16^ To address this limitation, researchers have focused on strategies to enhance lignin’s porosity, maximize its surface area, and improve the accessibility of binding sites, tailoring its properties to meet the demands of various applications.^17^ Modifying lignin to improve its adsorption performance is, therefore, a critical area of investigation. This can be achieved by introducing functional groups with high affinity for heavy metals through targeted chemical modifications. Additionally, surface activation methods, such as nanostructuring or the development of porous structures, have proven to be effective in increasing the surface area and creating more active sites for adsorption.

One particularly effective approach for making porous materials is the sol–gel process, a wet-chemical technique involving hydrolysis and polycondensation reactions of molecular or colloidal precursors that transition from a liquid “sol” into a solid “gel” phase. Upon drying, such gels can form a xerogel, a porous solid in which the solvent is removed by subcritical drying (typically at moderate temperatures and atmospheric pressure), in contrast to aerogels, which are dried under supercritical conditions, and cryogels, which are produced via freeze-drying. This method allows for precise control over structural properties, such as pore size, morphology, and surface area, enabling the design of porous materials tailored to specific applications.^18^

Furthermore, the versatility of the sol–gel process lies in its ability to incorporate phase separation dynamics, which can result in complex, hierarchically porous structures. By balancing the polymerization-induced phase separation (PIPS) with the sol–gel transition, it is possible to produce materials with a bicontinuous 3D network of interconnected pores.^19–21^ This structure often includes micro- and/or mesopores within a macroporous skeleton, creating a high surface area and multiple adsorption sites, which is a valuable trait for wastewater treatment applications. Originally focused on silica and organo-silicon materials, this approach has been adapted to other oxides, including titanium,^22^ zirconium,^23^ and aluminum,^24^ as well as organic networks such as phenolic resins, including resorcinol–formaldehyde^25^ and poly­(4-vinylphenol)–formaldehyde,^26^ demonstrating its versatility.

Herein, we used the sol–gel process to produce lignin-based xerogels (LBX) via the PIPS technique, taking advantage of the lignin/5-methylfurfural (5MF) reaction with poly­(ethylene glycol) (PEG) added for morphological control.

PIPS is a process characterized by the spontaneous separation of an initially homogeneous solution induced by the polymerization of one or more components within the system. While this process can occur in the presence of the reaction solvent alone, it may be further tuned through the addition of polymeric additives. The increase in molecular weight of the gelling components in the presence of an additive polymer induces mutual immiscibility, ultimately creating a greater driving force for phase separation via entropic effects. Following the onset of phase separation, the size of the phase-separated domains increases via a coarsening process. Consequently, the evolution of phase separation with respect to the sol–gel transition shapes the gel morphology. The larger the amount of time between the onset of phase separation and the sol–gel transition, the greater the characteristic size of the pores becomes. In this context, control over the structural characteristics and overall morphology of the xerogels may be achieved by varying the concentration and the molecular weight of the additive polymer used.^19^

Several recent studies have explored lignin- or polyphenol-based xerogels but with distinct feedstocks, chemistries, and objectives. Mikova et al. synthesized tannin–furfuryl and tannin–lignin–furfuryl organic xerogels by polycondensation, which were subsequently carbonized into carbon xerogels.^27^ They demonstrated that the tannin/furfuryl ratio and lignin incorporation strongly influenced porosity, particle size, and adsorption capacity, reporting benzene vapor uptake values of up to 0.83 g/g at 25 °C. De Moraes et al. developed cellulose/kraft lignin composite xerogels via dissolution and nitric acid coagulation, followed by drying and calcination.^28^ Their study highlighted how increasing the lignin content and calcination temperature enhanced microporosity and electrochemical activity, leading to improved H_2_O_2_ electrogeneration in gas diffusion electrodes. Moreira et al. produced bioxerogels directly from crude kraft black liquor blended with condensed tannin and cross-linked with furfural, glyoxal, or formaldehyde, using subcritical drying after solvent exchange.^29^ They systematically examined how tannin content, cross-linker type, and formulation ratios affected gelation, shrinkage, porosity, and mechanical properties, ultimately showing that furfural-cross-linked systems yielded more stable networks with porosity up to ∼49% and surface areas approaching 24 m^2^/g.

Our study offers a distinct perspective and differs from prior works in three key aspects: (i) feedstock, as we use a lignin-only network cross-linked with 5MF; (ii) approach to morphology control, where in situ PIPS is used to tune the morphology, going beyond the composition- and processing-driven approaches that dominate the current state of the art; and (iii) final state, by forgoing carbonization, thus reducing processing costs while maintaining the native chemical functionality of lignin posited to be relevant for applications such as heavy metal adsorption.

A comprehensive investigation was carried out to determine the impact of several parameters, including the type of lignin and the amount of cross-linker, additive polymer, and catalyst, on both the sol–gel reaction and the resulting morphology of the xerogels. This systematic analysis sought to elucidate the impact of these parameters on the final material properties. Based on these efforts, it was shown to be possible to tune the morphology of the synthesized LBX monoliths. The resulting macroporous structures exhibit promising levels of permeability, which are crucial for applications such as wastewater treatment. Additional post-treatment methods such as pyrolysis or etching, or alternatively, adjustments to the initial reaction composition, could be envisioned to increase microporosity and address the relatively low surface areas reported here with an eye toward greater adsorption capacity.

The implications of this work reach beyond the synthesis of porous materials, offering a sustainable and well-engineered pathway for transforming lignin, often regarded as industrial waste, into high-value LBX with a tunable morphology. It also reinforces the broader vision of integrating renewable resources into future technologies with promising relevance to applications such as heavy metal adsorption.

## 2 Experimental Section

### 2.1 Materials

Kraft lignin (KL), purchased from Sigma-Aldrich, reflects the typical structural features of industrial softwood Kraft lignins reported in the literature.^30^ Organosolv lignin (OL) was obtained from Chemical Point UG. Soda lignins, Protobind 2400 (SL1) and P6000 (SL2), were supplied by Tanovis AG. Endo-*N*-hydroxy-5-norbornene-2,3-dicarboximide was purchased from TCI Europe. 2-Chloro-4,4,5,5-tetramethyl-1,3,2-dioxaphospholane (TMDP), chromium III acetylacetonate, 5MF, *p*-toluenesulfonic acid (*p*TSA, 98.5%), sulfuric acid (95%), PEG with molecular weights of 400 g/mol, 1000 g/mol, 2000 g/mol, 3000 g/mol, 8000 g/mol, and 20000 g/mol, *n*-butanol (99.7%), cyclohexanol (99%), and dimethyl sulfoxide (DMSO, 99.9%) were all acquired from Sigma-Aldrich. Technical acetone (99%) was purchased from VWR, while methanol (99%) and *p*-dioxane (99.5%) were obtained from Carl Roth. Ethanol (95%), *n*-pentane (99.5%), dimethylformamide for gel permeation chromatography (DMF, GPC), and lithium bis­(trifluoromethanesulfonyl)­imide (LiTFSI, 99.9%) were purchased from Fisher Scientific.

### 2.2 Material Characterization

#### 2.2.1 Helium Displacement Pycnometry

The skeletal density ρ_*s*_ of the LBX was determined using helium displacement pycnometry (Accupyc II 1340, Micrometrics, Aachen, Germany) which operates on the principle of gas displacement to determine the volume of material present in the sample. ∼0.1 g of LBX was ground to powder, weighed, and analyzed.

#### 2.2.2 Bulk Density Measurements

The bulk density ρ_*b*_ of the LBX was determined using an envelope density analyzer (GeoPyc 1360, Micrometrics Ltd., Aachen, Germany) which operates on the principle of particle displacement to determine the exterior or envelope volume of the sample.

#### 2.2.3 Porosity Calculations

The porosity of the final dried LBX was calculated as follows (eq 1):

1 $$P_{\mathrm{total},\mathrm{dry}}\left(\mathrm{vol \%}\right)=\left(1-\frac{\rho_{b}}{\rho_{s}}\right)\cdot 100$$

#### 2.2.4 Gas Permeability

The gas permeability of the monoliths was determined by pressure rise measurements using a home-built device.^31^ In brief, LBX with a diameter of 12 mm were prepared as described, cut to a length of 25 mm, and then coated with rapid-curing epoxy resin Araldite RAPID. These coated samples were placed into a cylindrical PTFE mold with a diameter of 25 mm, after which the resin and hardener of a two-component epoxy system (Araldite 2020) were mixed and poured into the space between the sample and mold to seal the sample to avoid crossflow; the epoxy resin was then cured at 70 °C for 4 h (Figure 2).

**Figure 2:**
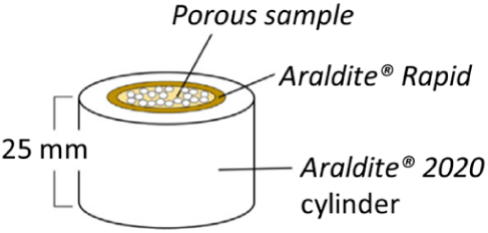
Schematic representation of the sample preparation for the gas permeability measurement. Reprinted with permission.^31^ Copyright 2020 Elsevier.

For each LBX sample, gas permeability was measured from both sides of the sample by increasing the inlet pressure from 0.1 to 2 Pa and taking ten individual measurements for each inlet pressure. After reaching constant pressure with the help of a vacuum pump (−0.6 Pa), nitrogen gas was passed through the sample, and the pressure rises were recorded. The permeability coefficient *K* was calculated as described by San Manley et al. (eq 2),^31^ where *Q*_2_ is the volumetric flow rate downstream, *P*_2_ is the downstream pressure, Δ*P* is the pressure difference across the samples, *A* is the cross-sectional area of the sample, *L* is the length of the sample, *P*_1_ is the initial pressure, μ is the fluid viscosity, *V* is the volume of the downstream chamber, *K*_0_ is the slip coefficient, *M* is the molecular weight of the permeant, *R* is the gas constant, *T* is the absolute temperature, and *t* is the time. The viscous permeability *K* was derived from the gradient of the linear plot of the permeability coefficient *K* vs *P*_*m*_ (mean pressure) (Figure S6).

2 $$K=\frac{Q_{2}P_{2}L}{\Delta PA}=\frac{V\left(\frac{dP_{2}}{dt}\right)L}{P_{1}A}=\frac{k}{\mu }P_{m}+\frac{4}{3}K_{0}\sqrt{\frac{8RT}{\pi M}}$$

#### 2.2.5 Micro-Computed X-ray Tomography (Micro-CT)

Micro-CT measurements were conducted to visualize and quantify in 3D the porosity of the materials. To this end, a laboratory X-ray cone-beam micro-CT system EasyTom 160 from RX Solutions (Chavanod, France) was used with the X-ray beam generated at 45 kV and 160 μA (small focus spot mode). 1440 projections over 360° (step of 0.25°) were recorded at 1 frame/s with a 16-bit flat panel imager (total pixel area of 1920 pixels × 1536 pixels). The source-to-object distance and the source-to-detector distance were set to ∼10 and ∼280 mm, respectively, providing a voxel size of ∼4.5 μm. The software XAct (RX Solutions, Chavanod, France) was used to reconstruct the sample volume into stacked slices from the recorded projections. During this reconstruction, the following corrections were performed: (i) automatic spot deviation correction to attenuate the effects of sample movement, (ii) image offset in one spatial direction to attenuate system misalignment effects, and (iii) a ring filter (set to 20 pixels) to attenuate the ring artifacts. The obtained stacked slices were then treated with 3D image analysis software Avizo (Konrad-Zuse-Zentrum Berlin/FEI SAS-Thermo Fisher Scientific, Waltham, MA, USA). To facilitate the segmentation and enhance the accuracy of analysis of the porous structure, the following procedure was applied: a median filter to attenuate image noise while preserving edges, gray-level segmentation of the pores, removal of insignificant small pores, and 3D quantification. The equivalent diameters of the pores were then calculated to estimate the pore size. At least three representative volumes of 250 × 250 × 250 voxels were considered for quantitative structural analysis. Results were reported as average values with standard deviations. To facilitate the 3D visualization of the pores, we employed the Avizo module thickness map. This tool provides a 3D representation of the largest sphere that can fit through each pore.

#### 2.2.6 Field Emission Scanning Electron Microscopy (FE-SEM)

FE-SEM was carried out on an SU-70 Hitachi microscope operating at 5 kV. Prior to analysis, LBX samples were sputter-coated with a 10 nm layer of platinum by using a Leica EM ACE600 Sputter Coater to improve their conductivity.

#### 2.2.7 Specific Surface Area (SSA) and Porosity Analysis

SSAs were measured via the Brunauer–Emmett–Teller (BET) approach using an Autosorb iQ gas sorption analyzer (Anton Paar/Quantachrome Instruments, Boynton Beach, FL, USA). 0.5–2 g of sample was degassed under vacuum at 100 °C for 2 days in 9 mm bulbless or 12 mm bulb borosilicate sample cells, depending on the sample quantity, with no insert rods, prior to measurement. Nitrogen gas adsorption–desorption isotherms were measured at 196 K, using a total of 40 data points for the adsorption and desorption branches. The relative pressure (P/P_0_) was gradually increased from 0.025 to 0.975 in uniform increments of 0.025, then increased a final time to 0.995, before decreasing back to 0.975, followed by increments of 0.025 in the same stepwise manner. At each pressure step, the system was allowed to equilibrate for 3 min. The multipoint BET method, using 11 data points within the relative pressure range of 0.050–0.3, was used to calculate the SSA. In cases where the correlation coefficient (*r*) was less than 0.99, the Micropore Assistant was used to refine the analysis, while the Barrett–Joyner–Halenda (BJH) method was applied to the desorption branch of the isotherm to assess the pore size distribution for pores smaller than ∼200 nm as well as the porosity associated with these pores specifically (referred to as “nanoporosity” in the context of this report).

#### 2.2.8 Thermogravimetric Analysis (TGA)

TGA of the LBX samples was performed from 50 to 800 °C using a TA Instruments Discovery TGA (Eschborn, Germany) with standard platinum pans, at a heating rate of 10 °C/min under a nitrogen flow of 25 mL/min.

### 2.3 Lignin/5MF System

In the first step, 1 g of each lignin (SL1, SL2, KL, and OL) was separately dissolved in a mixture of 4 mL of ethanol and 1 mL of water by vortex mixing for 1 min and then bath-sonicated (J. P. Selecta Ultrasons-HD) for 30 min. To this mixture, 5 g of 5MF was added along with 1 g of 8 M H_2_SO_4_ solution or 2 g of *p*TSA, followed by vortex mixing for 1 min. The mixture was cured in a 20 mL glass tube using a Radleys Carousel 12 Plus Reaction Station for 2 days at 90 °C. The resulting gels underwent solvent exchange with acetone in 500 mL Schott Duran borosilicate glass bottles under stirring, repeated until the acetone became colorless, to remove all the unreacted materials. Pentane was selected for the final solvent exchange step due to its ability to reduce the magnitude of the capillary pressure (*P*_c_) experienced by the xerogel during ambient drying, thus minimizing damage to the material and at least partially mitigating the collapse of the smaller pores. This pressure is influenced by several factors, including the surface tension of the fluid (γ), the contact angle between the fluid and the pore wall (θ), and the pore radius (*r*), as described in eq 3. The low RT surface tension of pentane (15.48 dyn/cm) compared to water (72.8 dyn/cm), ethanol (22.32 dyn/cm), or acetone (23.32 dyn/cm)^32^ minimizes the forces exerted on the gel network as the solvent evaporates, helping to preserve the pore structure and prevent collapse, especially in smaller pores. During solvent exchange, the gels experience deswelling-induced shrinkage as the reaction medium is replaced with pentane. This occurs because pentane is capable of mixing with acetone but, given its highly nonpolar nature, will not effectively swell the lignin–5MF network. As such, the presence of large quantities of pentane extracts the acetone, causing the polymer that makes up the gels to contract and rigidify prior to any drying. This loss of what is effectively a plasticizer improves the gel’s resistance to deformation caused by capillary forces. Furthermore, the lower compatibility between pentane and lignin–5MF also implies a higher contact angle, further reducing the capillary pressure. All of these factors make pentane an optimal solvent for minimizing drying-induced shrinkage and structural evolution. Finally, the very low boiling point of pentane favors complete solvent removal as well. To generate LBX, all gels were dried at ambient temperature for 1 night, followed by oven-drying for 1 week at 140 °C to be certain that no residual solvent of any type remained.

3 $$P_{c}=\frac{2\gamma \cos\theta }{r}$$

The reactivity of lignins, which influences their cross-linking potential during gel formation, was assessed by estimating the number of reactive sites per lignin molecule based on lignin unit composition and phenolic hydroxyl concentration derived from nuclear magnetic resonance (NMR) analysis, along with their molecular weights. The calculation method, resulting values, and molecular weights of the lignins are provided in Section 1 of the Supporting Information.

### 2.4 Lignin/5MF/PEG System

To investigate the effect of PEG molecular weight and content on gel formation, formulations were prepared using PEGs with molecular weights of 400, 1000, 2000, 3000, 8000, and 20000 g/mol, at varying PEG-to-lignin weight ratios (1:10, 1:1, and 10:1), while keeping the overall proportion of starting materials consistent across all samples, with OL selected as the basis for all networks formed in the remainder of the current study, following the preliminary screening of all lignin grades described previously. Reactions were carried out in 14 mL conical polypropylene centrifuge tubes. The water content was increased from 1 to 3 mL, as this adjustment was determined to promote macropore formation. For the highest PEG/OL ratio (10:1), the total reaction volume was reduced to accommodate tube capacity limitations. However, the relative proportions of the components were maintained in line with the other formulations (Table 1). All other steps in the protocol remained unchanged. The general LBX preparation procedure is illustrated in Figure 3.

**Table 1:** Typical Starting Compositions for OL–5MF Sol–Gel Reactions

PEG/OL ratio	OL (g)	5MF (g)	PEG (g)	*p*TSA (g)	EtOH (ml)	H_2_O (ml)	Estimated solids content (vol %)^a^	Estimated porosity (wet, vol %)^b^
1:10	1	5	0.1	2	4	3	40	60
1:1	1	5	1	2	4	3	37.5	62.5
10:1	0.33	1.66	3.33	0.66	1.33	1	24	76

a Calculated using the equation: $\Phi_{\mathrm{solids}\;\mathrm{content}}\left(\mathrm{vol \%}\right)=\left(\frac{V_{\mathrm{OL}}+V_{5\mathrm{MF}}}{V_{\mathrm{total}}}\right)\cdot 100$ .

b Calculated using the equation: $P_{\mathrm{estimated},\mathrm{wet}}\left(\mathrm{vol \%}\right)=\left(\frac{V_{\mathrm{total}}-\left(V_{\mathrm{OL}}+V_{5\mathrm{MF}}\right)}{V_{\mathrm{total}}}\right)\cdot 100=100-\Phi_{\mathrm{solids}\;\mathrm{content}}\left(\mathrm{vol \%}\right)$ .

**Figure 3:**
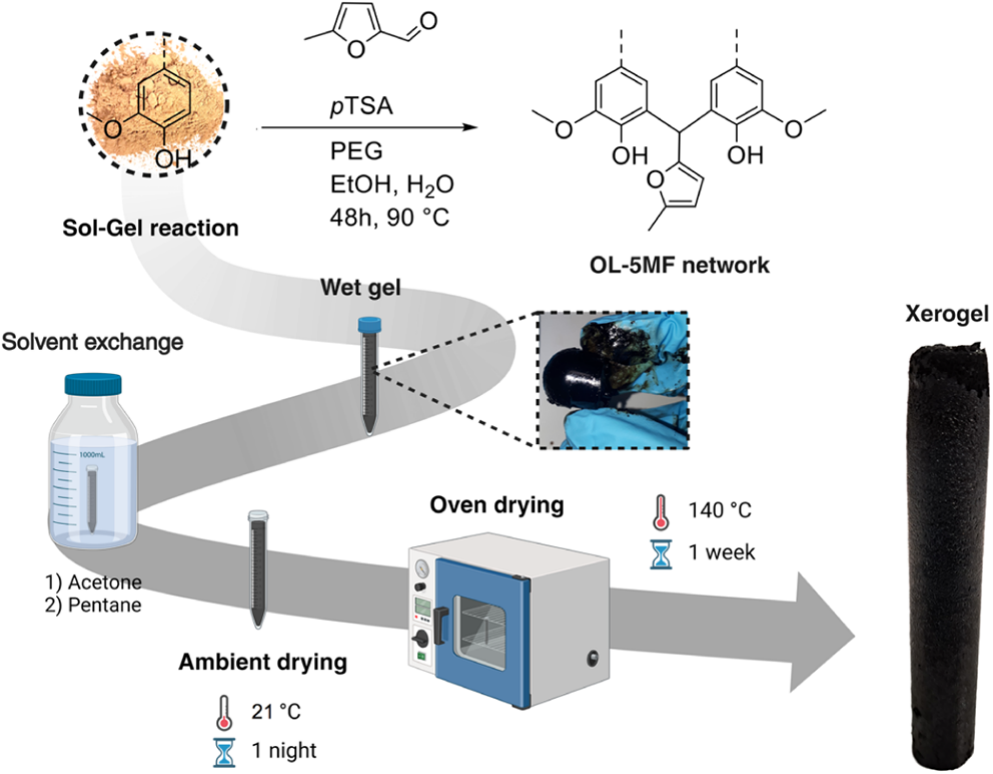
Schematic illustration of the general LBX preparation procedure.

## 3 Results and Discussion

### 3.1 Lignin/5MF System

#### 3.1.1 Optimization and Parameter Study of Sol–Gel Reaction Conditions

LBX were synthesized using a green, cost-effective approach via a resorcinol–furfural-type sol–gel reaction. In this system, lignin served as the primary aromatic component, while 5MF was selected as the cross-linker due to its lower toxicity compared to furfural or formaldehyde coupled with the fact that it may be readily derived from a range of biobased feedstocks.

The first research question to answer in this context concerns the optimal conditions for complete gelation. Here, a series of sol–gel reactions using SL1, 5MF, sulfuric acid, ethanol, and water were carried out using various component ratios and reaction times (Table S2). SL1 was selected as an appropriate test case for establishing baseline gelation conditions given that it is the least functional lignin that is nonetheless estimated to possess an average of more than two reactive sites per molecule (Table S1). This system was expected to be amenable to gel formation but only under well-optimized conditions (vs SL2, which is expected to gel poorly due to its low functionality, and KL and OL, which are expected to gel even at suboptimal conditions due to their high functionality). By this logic, the conditions effective for gel formation based on SL1 would be expected to be at least as effective for forming a gel with the more highly functional KL and OL. The cross-linking mechanism here is analogous to the traditional acid-catalyzed resorcinol–furfural system, as both involve electrophilic aromatic substitution followed by condensation reactions.^33^ Under acidic conditions, the aldehyde group of 5MF becomes highly reactive, facilitating electrophilic substitution primarily at the ortho positions of the aromatic rings in lignin. This reactivity is most prominent in the G and H units of lignin, as their ortho positions remain unoccupied, unlike in the S unit, where both ortho positions are blocked by methoxy groups. The reaction proceeds through the formation of hydroxymethyl intermediates, which undergo protonation and subsequent dehydration to yield methylene (−CH_2_−) bridges. The nature of the resulting cross-linked network depends on the density of reactive sites in the lignin and the reaction conditions. It was observed that achieving full gelation required 48 h, significantly longer than the 0.5 h reported in the literature for furfural.^34^ This extended gelation time can be attributed to the additional methyl group on the furan ring of 5MF, which decreases its reactivity and slows the kinetics of lignin cross-linking due to steric hindrance.

Once optimal conditions were identified, using SL1 as the lignin, sulfuric acid as the catalyst, and ethanol/water as the solvent mixture at 90 °C for 2 days, additional screening tests were conducted to examine the effects of different solvents, catalysts, and technical lignin types (Table S2). The sol–gel reaction worked well in the presence of lower alcohols, with ethanol and methanol yielding the best results. In contrast, incomplete gelation occurred with *n*-butanol and cyclohexanol, and no gelation was observed with dioxane or DMSO, despite their ability to effectively solubilize lignin. Protic solvents like ethanol and water appear to be more suitable, as demonstrated in the literature. The ethanol/water solvent system not only provides a homogeneous reaction medium but also actively reduces the energy barriers in the addition reactions through explicit solvent effects, thereby facilitating smoother and milder reaction conditions. Additionally, the protic nature of these solvents enables hydrogen bonding, which stabilizes intermediates and transition states, particularly through the formation of hexatomic ring structures.^35^ Furthermore, the polarity and high dielectric constants of such solvents enhance proton solvation, promoting greater ionization of the acid catalyst and thereby increasing the concentration of active protons in solution, which is essential for driving the condensation reaction with 5MF.^36^

The reaction proceeded only under acidic conditions, with *p*TSA and sulfuric acid identified as the most effective catalysts. Gelation was incomplete with HCl and absent with weaker acids, such as acetic acid, formic acid, and phosphoric acid. This behavior is attributed to the acid strength with strong acids catalyzing the reaction more effectively. It is noteworthy that gelation was incomplete with HCl, despite its higher acidity (p*K*_a_ = −6.3) compared to *p*TSA (p*K*_a_ = −1.3). This apparent inconsistency may be explained by the tendency of HCl to engage in side reactions with 5MF, given a report that shows HCl can react with aldehydes to form chlorinated derivatives.^37^ Such side reactions could limit the availability of 5MF for polymerization, thereby reducing the efficiency of HCl as a catalyst, despite its strong acidity.

The dimensional shrinkage (*S*_dim_) after solvent exchange and subcritical drying of the LBX samples was estimated using eq 4, assuming an isotropic reduction in volume, where *d*_1_ represents the LBX wet diameter before solvent exchange and *d*_2_ denotes the diameter after solvent exchange and drying, calculated as the average of the top and bottom diameters. The disadvantages of this approach are that it is approximate at best (*d*_1_ and *d*_2_ are measured to the nearest millimeter; given a typical wet diameter of *d*_1_ ∼ 13 mm, this implies a linear uncertainty of ∼10% and a volumetric uncertainty of ∼20%); it assumes that the gel is a perfect cylinder with a uniform diameter (whereas some variation in diameter is observed in practice; see Figure 4b); and it assumes that all shrinkage is perfectly isotropic in nature (further increasing the uncertainty). Nevertheless, it has the advantage of representing a direct measurement of the change in the volume of the LBX monolith and can provide a rough idea of the extent of shrinkage associated with the LBX formation process. The resulting estimates showed that *p*TSA-catalyzed OL-based xerogels (OLBX) exhibited a relatively low shrinkage compared to most other lignins, implying a higher cross-linking density and greater reactivity, in line with its higher estimated reactive site concentration than the other lignins (Table S1). This was further supported by high-temperature swelling of the *p*TSA-catalyzed OLBX in DMSO, which resulted in a gel fraction of 98% indicating a highly cross-linked OL–5MF network (Figure S7). Conversely, SL2-based LBX showed the highest shrinkage, consistent with its lower reactive site concentration and functionality per molecule than those of the other lignins (Table S1). These relationships highlight how differences in cross-linking behavior may be directly linked to variations in lignin structure. The *p*TSA-catalyzed LBX consistently demonstrated superior performance, achieving yields exceeding 90% across all samples (vs lower yields when H_2_SO_4_ was used), and exhibited a more homogeneous character compared to their H_2_SO_4_-catalyzed counterparts, showing a smoother appearance (Figure 4b) along with a more uniform microstructure (Figure 4c). While initially unexpected, these differences may be explained by several factors. First, condensation reactions between benzylic carbons (Cα) and the C6 position of another aromatic ring in the presence of sulfuric acid lead to cross-link formation through acid-induced hydrolysis, which could reduce its reactivity.^38,39^ Second, dilute sulfuric acid solutions have also been reported to induce furan-based aldehydes to form pseudolignins, especially when lignin is present.^40^ These side reactions and the generation of additional polymeric species may interfere with the gelation reaction and increase the heterogeneity of the system, explaining the observed results.

4 $$S_{\dim}\left(\mathrm{vol \%}\right)=\left[1-{\left(\frac{d_{2,\mathrm{dry}}}{d_{1,\mathrm{wet}}}\right)}^{3}\right]\cdot 100$$

**Figure 4:**
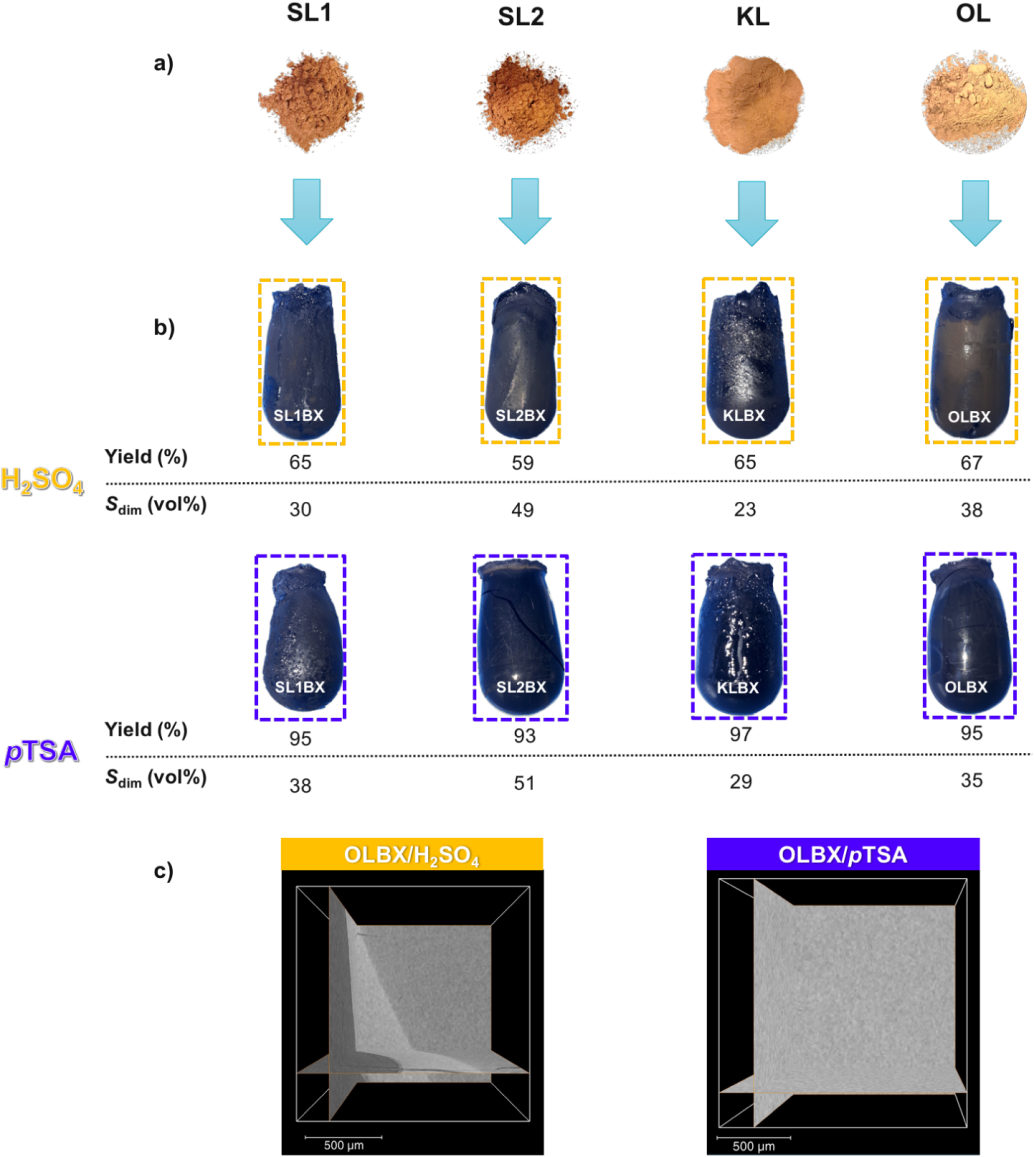
(a) Digital images of raw lignins (SL1, SL2, KL, OL); (b) LBX obtained using sulfuric acid and *p*TSA as catalysts in ethanol with 25 vol % water, with corresponding yield and *S*_dim_; and (c) orthogonal slices of OLBX obtained by micro-CT, comparing the effect of both catalysts on pore structure, with pores shown in dark gray.

Building on the aforementioned findings, OL and KL exhibited the highest concentrations of reactive sites with closely matching values, which was reflected in their gelation behavior. As shown in Figure 4b, both lignins achieved comparable gel yields when catalyzed by sulfuric acid (67% for OL and 65% for KL) and similarly reached yields of 95% and 97%, respectively, when using *p*TSA. Regarding *S*_dim_, OL and KL exhibited shrinkages of 38% and 23% with sulfuric acid, vs 35% and 29% with *p*TSA, respectively. However, visual assessment of homogeneity (Figure 4b) indicated that OLBX displayed greater homogeneity compared to KL, supporting its selection alongside *p*TSA as the optimal catalyst for further investigation in PEG-containing systems.

### 3.2 Lignin/5MF/PEG System

After optimizing the main parameters of the sol–gel reaction, including lignin type, catalyst, and solvent system, PEG was introduced to create hierarchical LBX with controlled macroporosity. PEG was selected as an additive polymer due to its excellent water solubility, commercial availability, and cost efficiency. Initially, the mixture is homogeneous, but as the molecular weight of the OL–5MF network increases due to cross-linking between OL and 5MF, its compatibility with PEG is expected to decrease. Reduced compatibility induces spinodal decomposition, resulting in simultaneous microscopic and/or macroscopic phase separation.^19^ The formation of bicontinuous domains is the logical outcome of such a process, with one phase as a wet gel and the other as a liquid. The morphology of LBX can be tailored by controlling the timing of phase separation relative to the sol–gel transition through variations in the composition and reaction parameters. With this in mind, the effects of the PEG molecular weight and concentration were investigated.

The initial hypothesis of this work was that the system in question could be considered analogous to the silica/poly­(ethylene oxide)/solvent (silica/PEO/solvent) system in terms of phase separation,^19,41^ where strong interactions between PEO and silica (e.g., hydrogen bonding, electrostatic attraction) drive the phase separation process. In the silica/PEO system, PEO adsorbs onto the silica surface, shielding the hydroxyl groups of the hydrophilic silica oligomers. This adsorption modifies the surface properties of silica, as the hydrophobic moieties of the PEO chains are exposed to the polar solvent, rendering the silica–PEO complex hydrophobic. Consequently, early phase separation occurs between the solvent phase and the silica–PEO gel phase.

In the OL/5MF system, in contrast, PEG was found to facilitate the dissolution of lignin (Figure S8) and to help maintain the growing OL–5MF network in the soluble state within the mixture. This delay in the onset of phase separation allowed the network to remain in solution longer, even if entropic effects are eventually expected to win out over enthalpic effects, resulting in eventual polymerization-induced phase separation. Beyond that, it was observed that increases in solvent (ethanol) content led to increased porosity (Figure S9), in line with expectations. However, the additional pores thus generated appear to be smaller than the detection limit of the tomographic technique applied here (∼3 μm).

The next hypothesis to be tested was the argument that an increased water content would favor phase separation and the generation of a broader pore size distribution, given that lignin’s relative hydrophobicity would tend to favor phase separation from aqueous media. An optimal water content of 75 vol % relative to ethanol was identified to induce this effect. Above this threshold, gelation became incomplete. The pore size distribution of the LBX was analyzed by using micro-CT imaging across different zones of the dried xerogels from multiple batches in order to assess the homogeneity and reproducibility of the samples. The results, presented in Table 2, show average pore sizes and porosities across the different conditions with one standard deviation indicated.

**Table 2:** Summary of LBX Sample Characterization, Including Skeletal Density, Pore Size, Porosity, Shrinkage, SSA, and Permeability, Measured Using Various Techniques^c^

	ρ_skeletal_ (g/cm^3^)	Pore size	Porosity (vol %)				SSA (m^2^/g)	Perm. (darcy)
Sample	He pyc.	micro-CT (μm)^a^	Env. density and He pyc.	BJH method^b^	*S*_dim_ (vol %)	*S*_por_ (vol %)	BET	(Δ*P*)
OLBX-400-0.1	1.3853	70 ± 40	65 ± 7	0.82	39	0	1.7	3.2 ± 0.5
OLBX-400-1	1.4141	70 ± 40	64 ± 6	0.28	39	0	0.8	2.8 ± 0.1
OLBX-400-10	1.3472	20 ± 10	73 ± 2	3.26	54	11	3.8	1.3 ± 0.05
OLBX-1000-0.1	1.3772	40 ± 20	59 ± 3	9.13	39	2	1.1	2.5 ± 0.01
OLBX-1000-1	1.3854	50 ± 20	67 ± 1	1.23	39	0	1.1	2.3 ± 0.2
OLBX-1000-10	1.3684	10 ± 10	69 ± 6	3.69	67	23	5.9	n.d.
OLBX-2000-0.1	1.3586	90 ± 50	62 ± 2	0.13	39	0	0.7	5.4 ± 0.9
OLBX-2000-1	1.2785	50 ± 25	44 ± 5	0.13	39	33	0.9	5.6 ± 0.3
OLBX-2000-10	1.3069	20 ± 10	49 ± 8	4.97	67	53	9.1	n.d.
OLBX-3000-0.1	1.4003	60 ± 30	60 ± 5	0.55	39	0	2.5	3.7 ± 0.1
OLBX-3000-1	1.3832	30 ± 20	56 ± 6	16	39	15	1.6	2.4 ± 0.1
OLBX-3000-10	1.2745	20 ± 10	19 ± 7	8.83	67	70	13.2	n.d.
OLBX-8000-0.1	1.382	30 ± 20	67 ± 8	0.55	39	0	0.7	2.7 ± 0.2
OLBX-8000-1	1.3983	30 ± 20	64 ± 2	0.97	39	0	1.5	3.3 ± 0.1
OLBX-8000-10	1.2875	10 ± 5	44 ± 14	8.37	77	57	4.4	n.d.
OLBX-20K-0.1	1.3558	30 ± 10	61 ± 3	0.94	39	0	5.2	3.2 ± 0.1
OLBX-20K-1	1.4038	30 ± 20	64 ± 9	0.42	39	0	0.8	n.d.
OLBX-20K-10	1.3414	30 ± 15	64 ± 6	2.10	77	33	2.6	n.d.

a Closed pores only.

b Calculated using the equation: $P_{\mathrm{nano},\mathrm{dry}}\left(\mathrm{vol \%}\right)=\left(\frac{V_{\mathrm{empty}\;\mathrm{space}}}{1+V_{\mathrm{empty}\;\mathrm{space}}}\right)\cdot 100$ , where *V*_empty space_ = *ρ*_skeletal_ · pore volume.

c n.d.: not determined, due to high sample shrinkage after drying.

As the PEG concentration increased, the skeletal density of the LBX samples, as measured through helium pycnometry, was observed to decrease. The relationship between the PEG concentration and the mechanical properties of the samples is consistent with this change. In the absence of PEG or at lower PEG concentrations, the samples were strong and brittle, while higher concentrations led to softer, weaker materials (Figure S10). This shift can be attributed to a decrease in cross-linking density within the solid network at elevated PEG concentrations. One explanation for this could be interactions between PEG molecules and lignin, which might restrict reactive functional groups and reduce their availability for cross-linking. The increase in solution viscosity at higher PEG concentrations may have hindered the mobility of the lignin and 5MF molecules as well. This interpretation aligns with the observed *S*_dim_ in the OLBX samples after solvent exchange and drying, where higher PEG content formulations experienced greater shrinkage compared to those with lower PEG content.

To assess the porosity of the samples, skeletal density measurements from helium pycnometry were combined with the envelope density data. This allowed for an accurate estimation of total porosity, which ranged from 19% to 73%, depending on the PEG molecular weight and PEG/OL ratio. The average porosity across all samples was found to be 59%. Notably, the OLBX-3000-10 sample exhibited a substantially lower porosity of 19%. This deviation is attributed to reductions in the wet strength of the gels at the highest PEG concentrations to the point where small changes in structure can result in large changes in their ability to resist drying-induced shrinkage, and highlights the opportunity to further optimize this approach.

The LBX samples displayed varying degrees of shrinkage after drying, which were primarily influenced by the PEG content. Samples with low or moderate PEG/OL ratios (1:10 and 1:1) exhibited constant *S*_dim_ values of approximately 39%. In contrast, samples with the highest PEG/OL ratio (10:1) showed more pronounced shrinkage, with *S*_dim_ values ranging from 54% to 77%, depending on the PEG molecular weight. Notably, OLBX-8000-10 and OLBX-20K-10 demonstrated the highest shrinkage with *S*_dim_ reaching 77% (Table 2). In addition to the reduced cross-linking efficiency implied by the skeletal density measurements, this aligns with the lower solids contents associated with systems with higher PEG content as well (Table 1), as lower solids content is typically associated with increased porosity and reduced mechanical resistance to capillary forces during drying. These conclusions are further supported by the observation of a logarithmic dependence of porosity on skeletal density (Figure S11), with the lowest skeletal densities associated with the lowest porosities (implying the highest levels of shrinkage).

Visually, the samples underwent a gradual color change from black to brown as the PEG content increased, likely due to the presence of growing concentrations of fine pores in the high-PEG samples (Table 1), where light scattering from the porous microstructure contributed to their lighter appearance, as shown in Figure 5. This visual shift correlates with structural differences in the pore size of LBX depending on the molecular weight and concentration of PEG used. For PEG 400, an increase in the PEG/OL ratio from 1:10 to 1:1 led to no significant changes in pore size (∼70 μm in both cases), but an increase to a PEG/OL ratio of 10:1 produced a substantial decrease in pore size to ∼20 μm at 10:1. Similar trends were observed with other PEG molecular weights with larger pore sizes associated with lower PEG/OL ratios and smaller pore sizes associated with higher PEG/OL ratios. For instance, with PEG 1000, the pore size decreased from ∼40 μm at 1:10 to ∼10 μm at 10:1, while with PEG 2000, the pore size decreased from ∼90 μm at 1:10 to ∼20 μm at 10:1. This trend persisted with higher molecular weights, such as PEG 3000, which yielded a pore size decrease from ∼60 to 20 μm as the PEG/OL ratio rose, and PEG 8000, where the pore size decreased from ∼30 to ∼10 μm. The exception to this trend was the highest PEG molecular weight (PEG 20K), where the pore size remained relatively stable at ∼30 μm, regardless of the PEG/OL ratio. This observation aside, these results align with the capacity of PEG at high concentrations to promote significant wet-state shrinkage driven by deswelling, thanks to a combination of the lower solids content and reduced cross-linking efficiency observed in the 10:1 PEG/OL samples.

**Figure 5:**
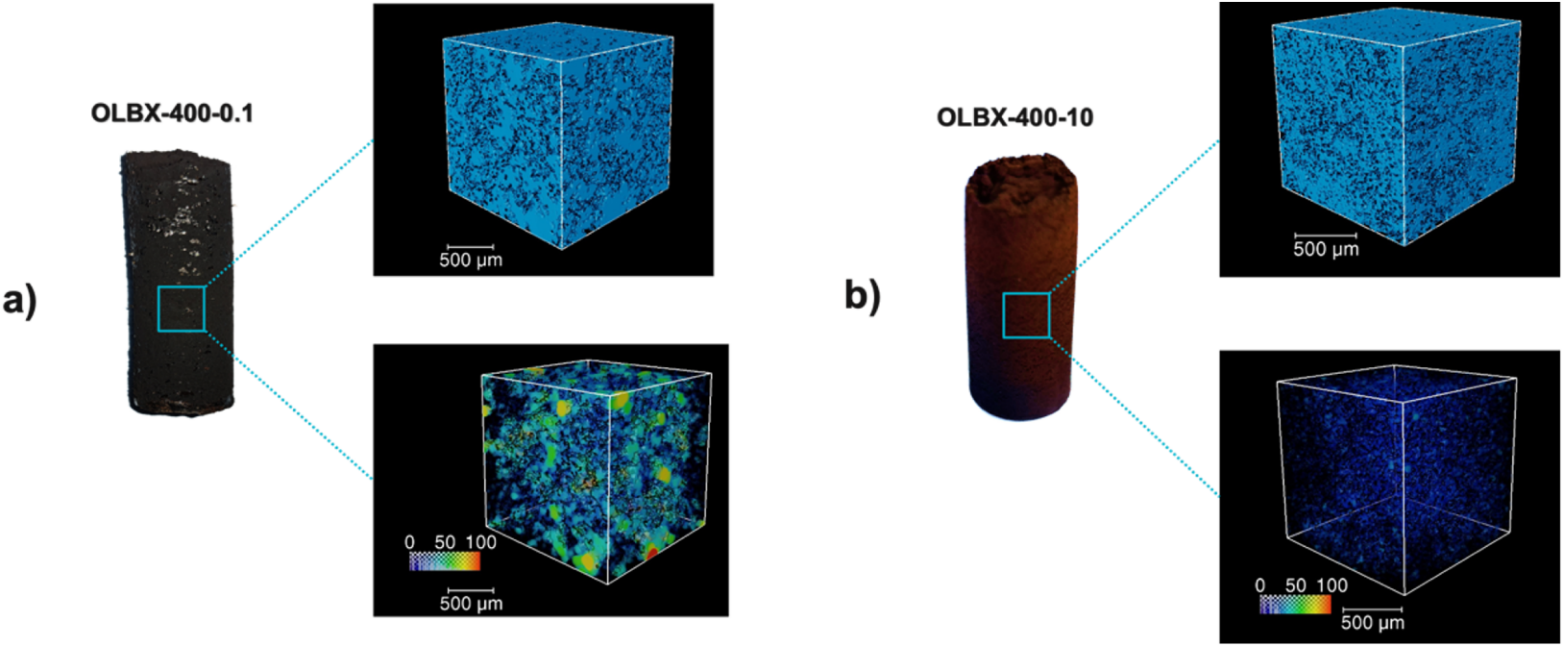
Visual appearance of selected LBX samples (a: PEG/OL ratio 1:10, b: PEG/OL ratio 10:1) and corresponding volume rendering of the pores (top) and pore thickness map analyses (bottom) obtained by micro-CT, illustrating the effect of increased PEG content on the pore size and porosity distribution. The color scale represents the diameter of the largest sphere fitting through a given pore, with blue, green, and red corresponding to 0 μm, 50 μm, and 100 μm, respectively.

Concerning the effect of PEG molecular weight on the pore size, it has been reported in silica sol–gel systems that higher molecular weight additive polymers induce earlier phase separation due to the “chemical cooling” concept, i.e., by reducing system entropy more effectively, favoring earlier phase separation during gelation.^20,21^ It follows that the onset of phase separation relative to the gelation point should vary with PEG molecular weight due to differences in entropic contributions, suggesting the possibility to control the structure of the porous network by tuning the molecular weight of the PEG. Based on the micro-CT results, however, there was no clear correlation between PEG molecular weight and pore size. This interpretation is complicated by the significant shrinkage observed in the 10:1 PEG/OL compositions, meaning that dry-state pore size measurements in these systems do not accurately represent the structure in the wet state. In contrast, less shrinkage was observed in the 1:10 and 1:1 PEG/OL compositions, providing a more reliable representation of the wet-state structure. Among these samples, PEGs with molecular weights between 400 and 3000 g/mol produced average pore sizes of approximately 60 μm, whereas higher molecular weight PEGs 8000 and 20000 g/mol resulted in smaller pores, averaging around 30 μm. While this seems counterintuitive at first, one possible explanation would be demixing of the larger PEG chains from the solution containing the OL–5MF network precursors even at low conversion levels. In this scenario, the PEG-rich phase no longer actively contributes to the coarsening process, which is critical for the development of larger domains. Consequently, the OL–5MF network cross-links within a confined and concentrated region, accelerating the sol–gel transition and accounting for the smaller pores observed compared to those formed with most lower molecular weight PEGs. While the intermolecular interactions at work in these systems are certainly different (and further work would be necessary to fully understand the complex behavior of the current system), this is broadly consistent with the shortened gelation times observed with increasing additive polymer molecular weight and concentration in silica sol–gel systems.^20^

Tomography analyses revealed significant variations in sample homogeneity, as indicated by the uniformity of the pore size distribution and the absence of macroscopic voids. Notably, higher PEG content generally led to denser and more homogeneous structures in the dry state (Figures S12–S13). This phenomenon is attributed to the substantial shrinkage caused by the lower solid content in the 10:1 PEG/OL compositions, effectively minimizing visible structural irregularities and producing the dense, uniform morphology observed in the dry samples. However, this does not exclude the possibility that these systems also exhibit a more homogeneous pore structure in the wet state. At high PEG concentrations (and lower PEG molecular weights), the observed ability of PEG to enhance lignin solubility in the initial reaction mixture may result in the delayed onset of phase separation, promoting a more uniform distribution of phase-separated domains throughout the mixture and enhancing structural uniformity, in addition to its impact on shrinkage.

Complementing the more approximate dimension-based shrinkage estimate (*S*_dim_), the total volume change experienced by the LBX samples due to solvent exchange and drying was also estimated via a porosity-based approach to the calculation of shrinkage (*S*_por_), as shown in eq 5. Here, the wet state porosity *P*_estimated,wet_ (estimated using the solids content; see Table 1) and the dry state porosity *P*_total,dry_ (measured using envelope density and helium pycnometry measurements; see eq 1) serve as the basis for the calculation. On its face, this approach would seem to be substantially superior to the dimension-based shrinkage estimates (*S*_dim_) described previously, given the much greater accuracy with which the wet state porosity may be estimated and the dry state porosity directly measured. However, this approach has its disadvantages, as well, given the need to make two key assumptions. First, it is assumed that the initial solids content is the only factor that determines the volume of material present in the LBX; for this to be true, the reaction yield must be 100% regardless of composition, whereas in practice, even in the absence of PEG, the yield is always at least slightly lower (see Figure 4b). Second, it is assumed that the process of converting the OL and 5MF in the initial reaction mixture to a solid OL–5MF network results in no change in mass or volume, whereas in reality some level of cross-linking-induced shrinkage will occur, in addition to which cross-linking will produce small molecule byproducts as it proceeds, meaning some losses in volume and mass are inevitable. All of these factors favor the realization of a higher porosity in the dry state than would otherwise be expected, given the assumptions made, resulting in an underestimation of the actual shrinkage. Unfortunately, given the potential for multiple different cross-linking reactions generating multiple byproducts, the lack of data on the reaction-induced shrinkage in such networks, and the potential for PEG addition to alter both tendencies, it is not possible to accurately correct for these losses. The consequence of these complications is that *S*_dim_ more accurately reflects the true shrinkage but with rather poor precision, while *S*_por_ provides greater precision at the expense of accuracy. This is reflected in the discrepancies between *S*_dim_ and *S*_por_ (Table 2), with the latter tending to give a lower value than the former, entirely in line with the above discussions. Nonetheless, the resulting heatmaps (Figure 6) illustrating the relationship between PEG molecular weight, PEG/OL ratio, and total shrinkage as assessed via both measures, as well as the average pore size of the dry xerogels, provide clear evidence of the same global trends in both shrinkage measurements. This visualization is crucial for distinguishing between systems that experience minimal shrinkage, where micro-CT and FE-SEM data more accurately represent the wet state structure, and those with significant shrinkage, where dry state imaging may not capture the true porosity and pore structure of the wet state. Taken as a whole, these results reinforce the conclusion that the systems containing the most PEG display the most shrinkage and the smallest final pore sizes.

5 $$S_{\mathrm{por}}\left(\mathrm{vol \%}\right)=\left(\frac{P_{\mathrm{estimated},\mathrm{wet}}-P_{\mathrm{total},\mathrm{dry}}}{1-P_{\mathrm{total},\mathrm{dry}}}\right)$$

**Figure 6:**
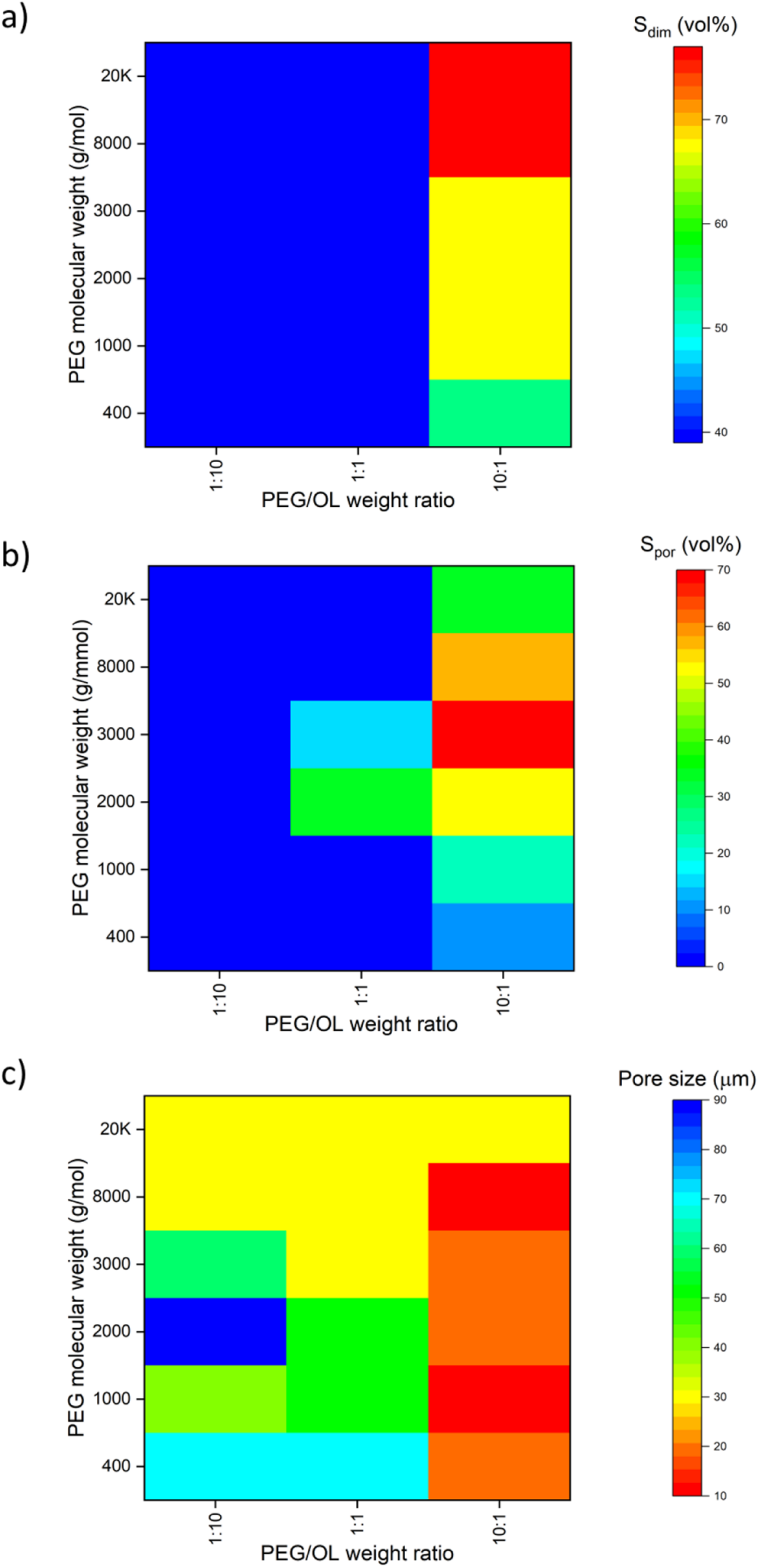
Heatmaps illustrating the effect of PEG molecular weight and PEG/OL weight ratio on (a) the geometry-based shrinkage *S*_dim_, (b) the porosity-based shrinkage *S*_por_, and (c) the resulting pore size of the prepared LBX materials.

To further examine the morphological characteristics and internal structure of LBX as influenced by PEG concentration and molecular weight, FE-SEM imaging was conducted (Figure 7). At lower PEG concentrations, the system formed larger aggregates. Conversely, at higher PEG concentrations, smaller aggregates were observed, which were embedded within a more compact network. This is primarily due to the low solids content in the high-PEG samples, which leads to considerable shrinkage upon solvent exchange and drying, resulting in densification of the pore network.

**Figure 7:**
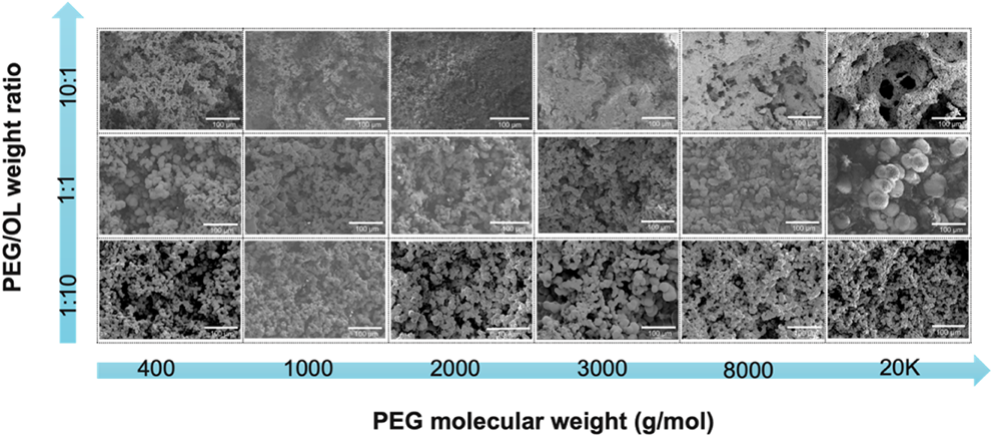
FE-SEM images illustrating the variation in LBX morphology as a function of the PEG molecular weight and PEG/OL weight ratio.

Moreover, the timing of gelation relative to phase separation plays a critical role in determining the size of the aggregates and macropores. Early phase separation allows coarsening to proceed, resulting in larger pores and aggregates. This tendency is evident in the SEM images, which show that at lower PEG concentrations, the xerogels exhibit coarser morphologies with larger globular clusters. However, as the PEG concentration increased, the aggregates became progressively smaller due to the delayed phase separation, which limited the time available for domain coarsening and resulted in finer, more densely packed globular clusters. Moreover, the final morphology of the phase-separated domains is governed by interfacial energy dynamics, which remains initially stable for a period until the gelation process “freezes” the growing network. However, in the case of slow gelation where phase separation occurs prior to the sol–gel transition, the domains fragment to minimize interfacial area and energy, eventually leading to isolated domains. This results, in our case, in the formation of microgel particles that continue to react, forming chain-like aggregates of globular particles, which is consistent with the observed morphology across all samples.

To complement the aforementioned methods and provide a more comprehensive analysis of the porosity, nitrogen sorption measurements were conducted to quantify the contributions from micro- and mesopores, which are beyond the detection limits of both tomography and envelope density techniques (Figure 8a; Table 2). The results revealed a limited concentration of pores with diameters less than ∼100–200 nm and SSAs in the range of ∼0.7–13.2 m^2^/g. These values align with the predominantly macroporous nature of the xerogels and are logical given the significant structural contribution of macropores. The relatively high porosity associated with small pores in high-PEG compositions can be attributed to significant shrinkage during deswelling, which reduces pore sizes and increases the microporosity, ultimately leading to higher SSA values (Figure 8b). However, the overall SSA remains relatively low. To enhance the adsorption potential of these xerogels, post-treatment strategies could be explored. Techniques such as pyrolysis or chemical etching may be employed to introduce additional micro- or mesopores, thereby increasing the total surface area. Alternatively, further reducing the solid content could induce greater shrinkage, promoting the formation of more fine pores and enhancing SSA.

**Figure 8:**
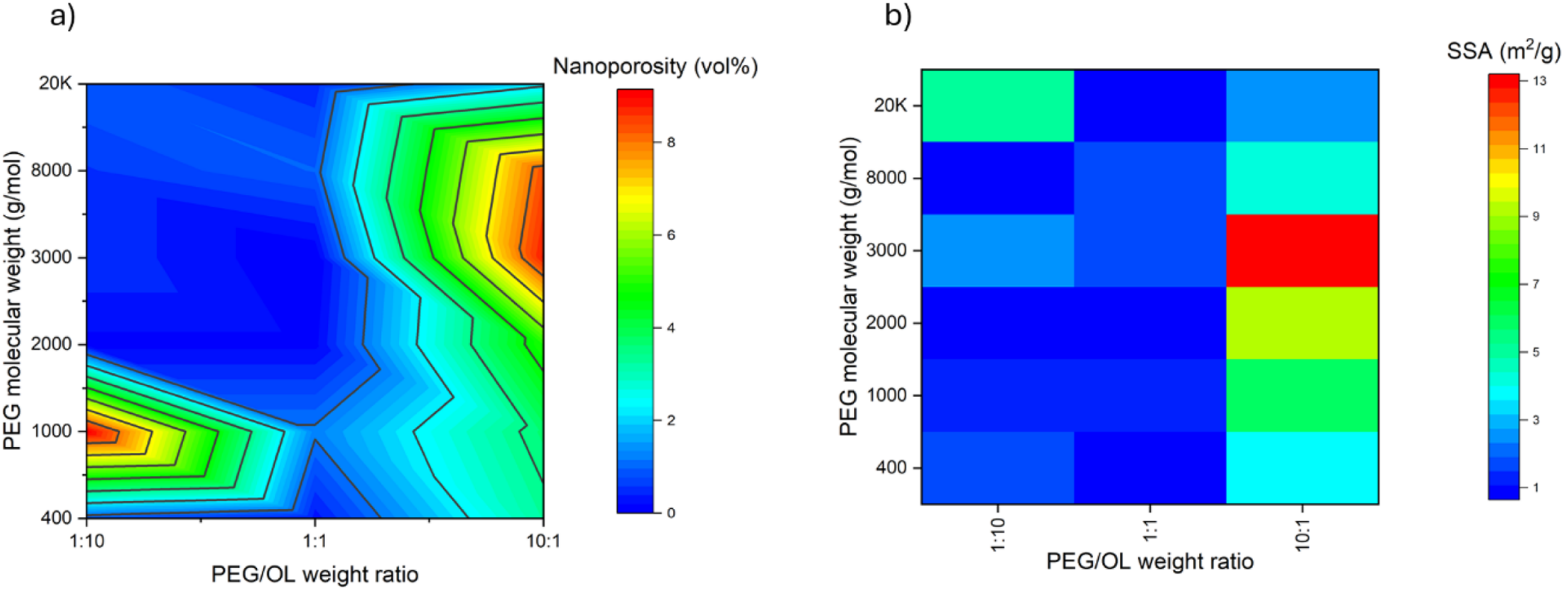
(a) Nanoporosity as estimated via the BJH technique as a function of PEG molecular weight and PEG/OL weight ratio. (b) Heatmap illustrating the effect of PEG molecular weight and PEG/OL weight ratio on the BET SSA of the prepared LBX materials.

With the morphology and porosity characterized, TGA was performed to determine decomposition temperatures and char yield in a nitrogen atmosphere, revealing distinct thermal degradation profiles that varied with both PEG molecular weight and PEG/OL ratio (Figure 9). These differences reflect the influence of PEG on the structure of the materials generated under each set of conditions, given that porosity and surface-to-volume ratio are expected to govern heat and mass transfer during decomposition and strongly impact the thermal stability of the xerogels.

**Figure 9:**
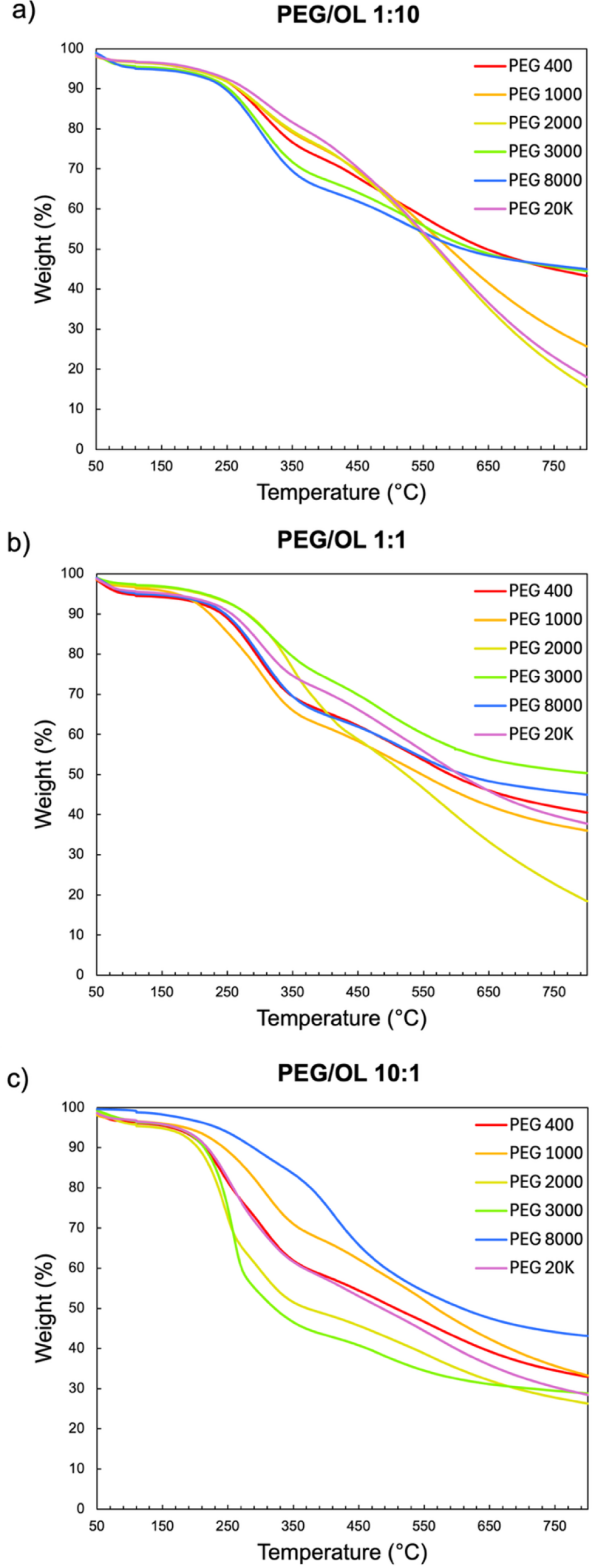
TGA curves of xerogels prepared with PEG/OL ratios of (a) 1:10, (b) 1:1, and (c) 10:1.

At low and intermediate PEG/OL ratios (1:10 and 1:1), the degradation profiles for different PEG molecular weights largely overlap, suggesting that PEG molecular weight has a negligible impact on the thermal response under such circumstances, likely due to similar structural characteristics across these compositions. In contrast, at the highest PEG/OL ratio (10:1), the influence of PEG molecular weight became more pronounced, resulting in a broader divergence of the degradation curves, with some samples exhibiting abrupt mass loss and others undergoing more gradual decomposition. The temperatures at the onset of decomposition highlight this trend. For the 1:10 PEG/OL xerogels, decomposition began within a narrow range of ∼245–260 °C. At 1:1, the interval broadened to ∼210–260 °C, and at 10:1 it shifted to ∼200–250 °C. This shift is posited to reflect increasing heterogeneity in the pore structure (Table 2), consistent with prior observations concerning greater variability in the shrinkage behavior of such systems. Considering these results in terms of structural parameters, the relatively similar porosity values observed in the 1:10 PEG/OL compositions (59–67%) yielded similar degradation profiles, while the broader distribution of porosities in the 1:1 PEG/OL compositions (44–67%) and especially the 10:1 PEG/OL compositions (19–73%) resulted in more variable thermal behavior, with the higher SSAs in the latter case posited to favor thermal degradation as well. Such differences in structure can facilitate or hinder the release of volatile products, thereby accelerating or delaying weight loss.

Char yields also varied with the PEG/OL ratio and PEG molecular weight. In the 1:10 PEG/OL compositions, PEG 400, PEG 3000, and PEG 8000 gave relatively high residues (43–45%), whereas PEG 1000, PEG 2000, and PEG 20K produced notably lower yields (26%, 16%, and 18%, respectively). Among the factors contributing to this behavior is their relatively low skeletal density (Table 2), which indicates a less compact framework that favors the formation of volatile degradation products over stable carbonaceous residues. A similar trend was observed in the 1:1 PEG/OL compositions, where most samples yielded 36–50% residue, but PEG 2000 again showed markedly lower char yield (18%), consistent with its particularly low skeletal density within this set. For the 10:1 PEG/OL compositions, the char residues fell within a narrower interval of 26–43%. When the relationship between structural parameters (porosity, SSA, and skeletal density) and measures of thermal degradation behavior (temperature at the onset of degradation, temperature of maximum degradation, and char yield) is analyzed across all of the xerogels produced, however, no clear correlations are observed, with most xerogels giving values in a relatively similar range and with variations arising from other factors otherwise not accounted for (for example, the absolute size and form of the TGA sample itself). These observations indicate that, overall, these materials are more similar than they are different, supporting the argument that the polymer network on which these xerogels are based is generally rather consistent in terms of structure and properties.

It is worth comparing the above results with the thermal degradation behavior of the raw lignin from which these xerogels are derived (Figure S5c). The onset of degradation of the raw lignins studied here occurs in the ∼210–260 °C range as well, fully consistent with what is observed for these xerogels. That the raw lignins lose most of their mass at temperatures higher than that of the xerogels is not surprising given that the 5MF-derived cross-links are liable to be less thermally stable in the latter case. In spite of this, however, char yields similar to or greater than that of the raw lignins (34–43%) may be realized in most cases. While an in-depth study of this behavior is beyond the scope of the current report, this nonetheless highlights the potential of this approach to generate precursors for porous carbons, given the increased carbonization yield implied by this observation.

In contrast, a comparison of differential scanning calorimetry (DSC) data between the parent lignins (SL1, SL2, KL, and OL) and the xerogels is less informative. While the former display clear glass transition temperatures (*T*_g_s), the corresponding cross-linked xerogels do not (Figure S5a,b). This is readily explained by the high cross-link density of these materials, which is known to broaden and weaken the glass transition.^42^

As the final step in characterizing the LBX samples, the permeability was evaluated, providing direct insight into the impact of pore architecture on fluid transport. The LBX exhibited high permeability values, ranging from 1.3 to 5.6 darcys, a promising result given the need for effective fluid flow through the porous material in order to realize optimal mass transfer and adsorption kinetics. This is in contrast with previously described polyepoxide foams produced via the high internal phase emulsion approach, which exhibited significantly lower permeability (20–200 mD) in spite of broadly similar levels of overall porosity and pore size—thus highlighting the impact of pore connectivity as well.^43^ Figure 10 illustrates the relationship between permeability, pore size, and porosity for the LBX systems. A general increase in permeability is observed as pore size increases, as expected, given that larger pores are anticipated to facilitate fluid flow by reducing resistance and enhancing permeability. In contrast, permeability appears to actually decrease somewhat with increasing porosity—an observation that can only be explained by changes in pore connectivity.

**Figure 10:**
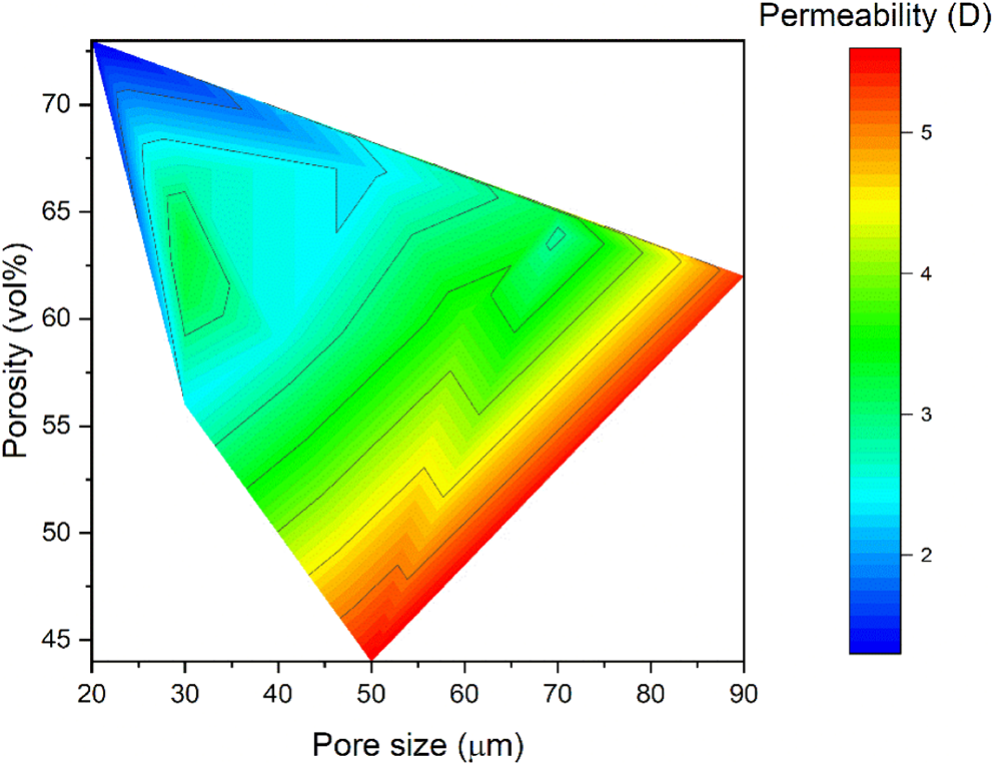
Permeability of LBX materials as a function of the pore size and porosity.

## 4 Conclusion

This study addressed the pressing need for sustainable wastewater treatment solutions by exploring the valorization of lignin, a widely available and underutilized biomass resource, to produce porous materials capable of removing heavy metals from wastewater. The objective was to develop and understand the formation of LBX with tailored porosity and morphology to enhance its environmental application potential.

The synthesis of LBX was achieved through the combination of an organic sol–gel process and PIPS, yielding macroporous materials with hierarchical structures. Characterization of the lignin structure and molecular weight directly predicted the network formation potential and the gravimetric yield of the sol–gel process. The incorporation of PEG as an additive polymer in this process played a crucial role in controlling the phase separation dynamics, pore morphology, and porosity. While PEG content had minimal impact on pore architecture at lower molecular weights (400–3000 g/mol), a distinct shift toward finer pores was observed beyond the 3000 g/mol threshold. Higher PEG concentrations, associated with lower solids content, led to greater isotropic deswelling-induced shrinkage, resulting in reduced pore sizes and increased SSA, as confirmed by micro-CT, BJH, and BET analyses. The resulting LBX materials exhibited favorable permeability, making them promising candidates for water treatment applications. Their relatively low SSA suggests that post-treatment modifications or compositional adjustments could be explored to introduce additional micro- and mesoporosity, thereby enhancing their adsorption capacity for heavy metals. At the same time, TGA demonstrates the potential to realize increased char yields vs the parent lignin, highlighting some additional benefits of this approach.

## Supplementary Material



## Notes

Characterization of the technical lignins (GPC, ^1^H NMR, ^31^P NMR, 2D HSQC NMR, TGA, and DSC); sol–gel reaction screening experiments; permeability coefficient of LBX as a function of mean pressure; gel fraction determination; digital images illustrating the enhanced solubility of OL in PEG/OL mixtures at a 10:1 ratio; orthogonal slices of LBX prepared under different solvent compositions, with corresponding FE-SEM images; digital images illustrating the PEG addition effect on the mechanical properties of the LBX; the correlation between OLBX porosity and skeletal density; tomography images alongside pore maps of LBX; large-scale orthogonal slices of LBX (PDF)
