# Supplementary material for: Tailored Lignin Xerogels: Insights into Morphology Control
Source: ACS Sustain Chem Eng. 2026 Mar 19;14(13):6291–304. doi: 10.1021/acssuschemeng.5c11885 (PMC13063406; doi:10.1021/acssuschemeng.5c11885)
Supplement: Supplementary file 1 [file sc5c11885_si_001.pdf]

Tailored Lignin Xerogels: Insights into Morphology Control

Aymane El Bouhali,[†,‡] Frédéric Addiego,[†] Hande Barkan-Öztürk,[§] Alexander Bismarck,[§,||] Jean-Sébastien Thomann,[†] Daniel F. Schmidt[†,]*

[†]Department of Materials Research and Technology, Luxembourg Institute of Science and Technology, L-4362 Esch-Sur-Alzette, Luxembourg

[‡]Department of Physics and Materials Science, University of Luxembourg, L-4365 Esch-sur-Alzette, Luxembourg

[§]Institute of Material Chemistry and Research, Faculty of Chemistry, University of Vienna, Währing Strasse 42, 1090 Vienna, Austria

[||]Department of Chemical Engineering, Imperial College London, South Kensington Campus, London, SW7 2AZ, UK

This Supporting Information document consists of 20 pages, including 13 figures and 2 tables.

1. Lignin

1.1 Characterization Methods

Gel Permeation Chromatography (GPC). GPC was performed on an Agilent 1200 CPL system (Agilent technologies, Craven Arms, United Kingdom). The chromatograph was equipped with an integrated IR detector, and 2 × PLgel 5 µm MIXED-C 5 300 × 7.5 mm columns. 0.1 M LiTFSI in DMF was used as the eluent to minimize intermolecular interactions and reduce adsorption on the GPC column, operating at a flow rate of 1.0 mL/min at 40 °C. Poly(methyl methacrylate) standards (EasiVial PMMA, Agilent Technologies) with peak molecular weights (M_p) ranging from 535 g/mol to 1,591,000 g/mol were used to perform the calibration of the system.

Nuclear Magnetic Resonance (NMR). NMR spectra were recorded on a Bruker AVANCE III 600 MHz spectrometer (Bruker, Fällanden, Switzerland) at 25 °C. For ^{1}H NMR, 15 mg of lignin was dissolved in 0.6 mL of DMSO-d_6. The acquisition parameters were as follows: 12,019 Hz spectral width; 128 scans; 2.7 s acquisition time; and 10 s relaxation delay. For 2D HSQC NMR, 200 mg of lignin was dissolved in 0.6 mL of DMSO-d_6. The acquisition parameters included 24 scans, with a spectral width of 25,641 Hz and an acquisition time of 5.6 ms for the F1 dimension, as well as a spectral width of 7,212 Hz and an acquisition time of 170 ms for the F2 dimension. Quantitative ^{31}P NMR was done following the procedure developed by Granata and Argyropoulos et al. Chromium (III) acetylacetonate and endo-N–hydroxy–5–norbornene–2,3 dicarboximide were used as the relaxation reagent and the internal standard (IS), respectively.

Differential Scanning Calorimetry (DSC). DSC thermograms were recorded on a Mettler Toledo DSC 3+ instrument (Mettler Toledo, Greifensee, Switzerland), in 40 µL standard pierced aluminum crucibles. The samples were heated and cooled under a nitrogen atmosphere using the following thermal cycles. First heating–cooling cycle: from –50 to 200 °C and from 200 to –50 °C at a rate of 10 °C/min, to remove residual traces of moisture and erase the thermal history of the material. Second heating cycle: from –50 to 200 °C at a rate of 10 °C/min. The glass transition temperature (T_g) was recorded as the midpoint temperature of the heat capacity transition observed during the second heating cycle.

Thermogravimetric Analysis (TGA). TGA of raw lignins was performed on a Mettler Toledo TGA 2 instrument (Mettler Toledo, Greifensee, Switzerland), in 70 µL standard alumina pans from 25 to 800 °C under a nitrogen atmosphere, at a rate of 10 °C/min.

1.2 Results

GPC was used in assessing the number average molecular weight (M_n), the weight average molecular weight (M_w), and the dispersity (Đ) of the lignin samples (Fig. S1). KL exhibited the highest M_n (1,090 g/mol), M_w (6,660 g/mol), and dispersity (Đ = 6.1). In contrast, SL2 had the lowest dispersity (Đ = 4.3), with M_n = 740 g/mol and M_w = 3,200 g/mol, while SL1 had similar molecular weights to SL2 but a slightly higher dispersity (Đ = 4.5). Finally, OL showed intermediate values (M_n = 810 g/mol, M_w = 4,520 g/mol, Đ = 5.6).

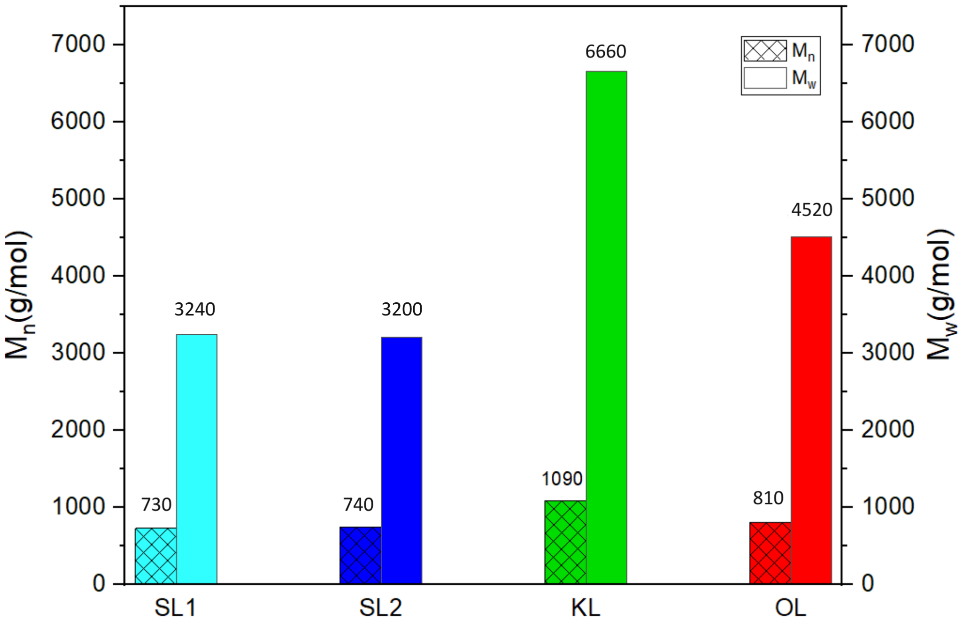

Figure S1. M_n and M_w determined by GPC of raw lignins.

NMR spectroscopy was used to analyze the structural composition and identify the functional groups present in the lignins. The ^{31}P NMR spectra of the phosphitylated lignins are presented in Fig. S2. Aliphatic alcohols, 5-substituted phenols, guaiacyl hydroxyls, *p*-hydroxyphenyl hydroxyls, and carboxylic acid groups present in the lignins were quantified by ^{31}P NMR after phosphorylation with TMDP using endo-N–hydroxy–5–norbornene–2,3 dicarboximide as an internal standard (IS). The results of OH groups analysis of the different lignins determined via quantitative ^{31}P NMR measurements are illustrated in Fig. S3. OL and KL had the highest aliphatic hydroxyl contents at 3.74 and 3.86 mmol/g, respectively, compared to SL1 (2.52 mmol/g) and SL2 (2.16 mmol/g). Aromatic hydroxyl groups were most abundant in OL (7.34 mmol/g), followed by KL (5.67 mmol/g), SL1 (5.5 mmol/g), and SL2 (3.0 mmol/g). OL also had the highest carboxylic acid content at 3.38 mmol/g, while KL exhibited the lowest at 0.45 mmol/g. The 2D HSQC NMR spectra are given in Fig. S4, with peak assignments established based on previous publications.[1-4] The lignin samples exhibited distinct variations in their S, G, and H unit distributions. SL1 showed a balanced composition with 49% G, 47% S, and 4% H units, while SL2 was G-rich (56% G, 43% S, 1% H). KL was mainly composed of G units (98%), with minimal H units (2%) and no S units. In contrast, OL featured a mixed structure with 67% G, 24% S, and a higher proportion of H units (9%). The monomeric composition determined by 2D HSQC NMR was further used to estimate the potential reactivity of the lignins based on the availability of reactive ortho positions on their aromatic units, which influences their capacity for crosslinking. The S units were considered unreactive due to the substitution of both ortho positions, while G units contained one available ortho site, and H units offered two. The number of reactive ortho positions per phenolic hydroxyl group (Ar–OH) was estimated according to Eq. S1. This theoretical reactivity index was combined with the quantitative ^{31}P NMR-derived Ar–OH content to calculate the total concentration of reactive ortho sites per gram of lignin. Finally, the product of the reactive site concentration and the number average molecular weight (M_n) yields an estimate of the number of reactive sites per lignin molecule, which is of critical importance for gel formation (where a value greater than two is required to yield a network). The resulting values, shown in Table S1, provide an estimation of the relative crosslinking potential of the lignins, based solely on ortho

site availability. OL and KL exhibited the highest concentrations of reactive ortho sites, suggesting a greater potential for crosslinking with 5MF. SL2 and SL1 (which has twice the reactive site content of SL2) possess fewer reactive sites vs. OL and KL and were therefore expected to exhibit lower reactivity. SL2 in particular has so few reactive sites per molecule that its ability to form a network is entirely dependent on the fact that its molecular weight distribution is broad enough that some higher functionality species should nonetheless be present.

$$\text{Reactive sites per Ar} - \text{OH} = 2(\%\text{H}) + 1(\%\text{G}) + 0(\%\text{S}) \qquad (\text{S1})$$

Table S1. Estimated number of reactive sites per phenolic hydroxyl and their concentration in different lignins.

Lignin	% H	% S	% G	Reactive Sites (per Ar–OH)	Ar–OH (mmol/g)	Reactive Sites (mmol/g)	Reactive Sites (per molecule)
SL1	4	47	49	0.57	5.5	3.1	2.3
SL2	1	43	56	0.58	3.0	1.7	1.3
KL	2	0	98	1.02	5.67	5.7	6.2
OL	9	24	67	0.85	7.34	6.2	5.0

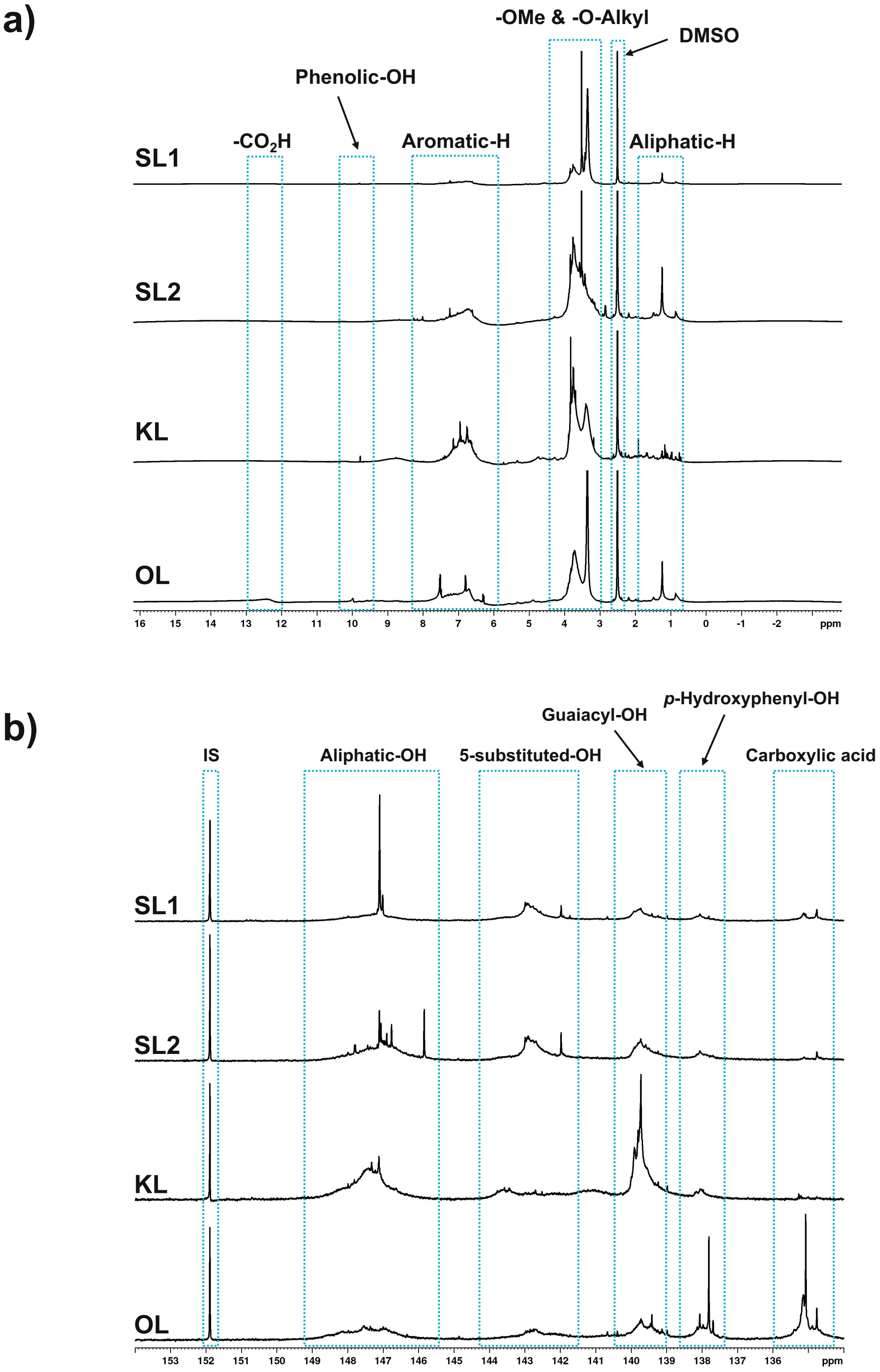

Figure S2. ^{1}H NMR (a) and ^{31}P NMR (b) spectra of raw lignins.

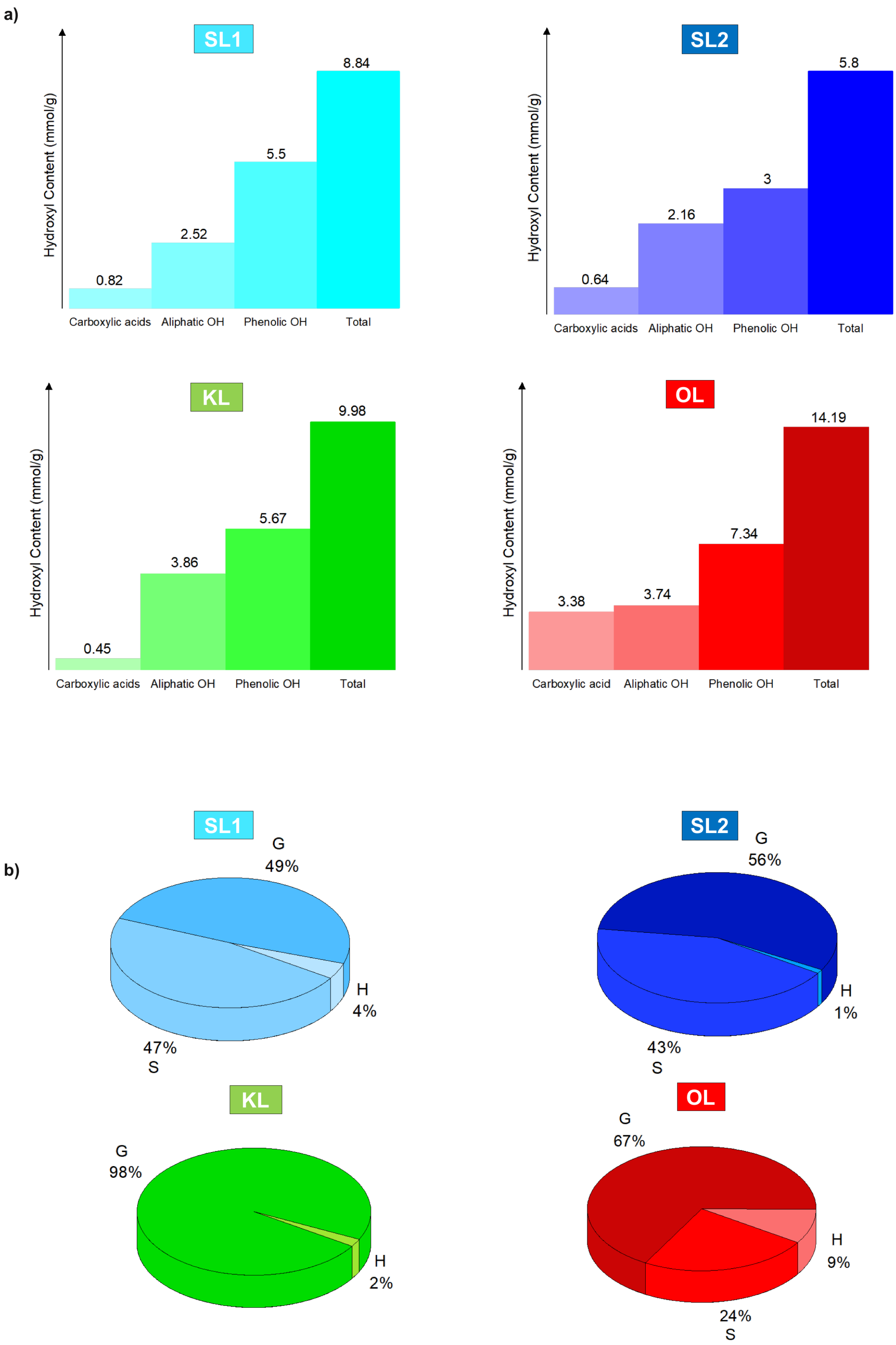

Figure S3. OH content determined by ^{31}P NMR and SGH composition analyzed via 2D HSQC NMR of raw lignins.

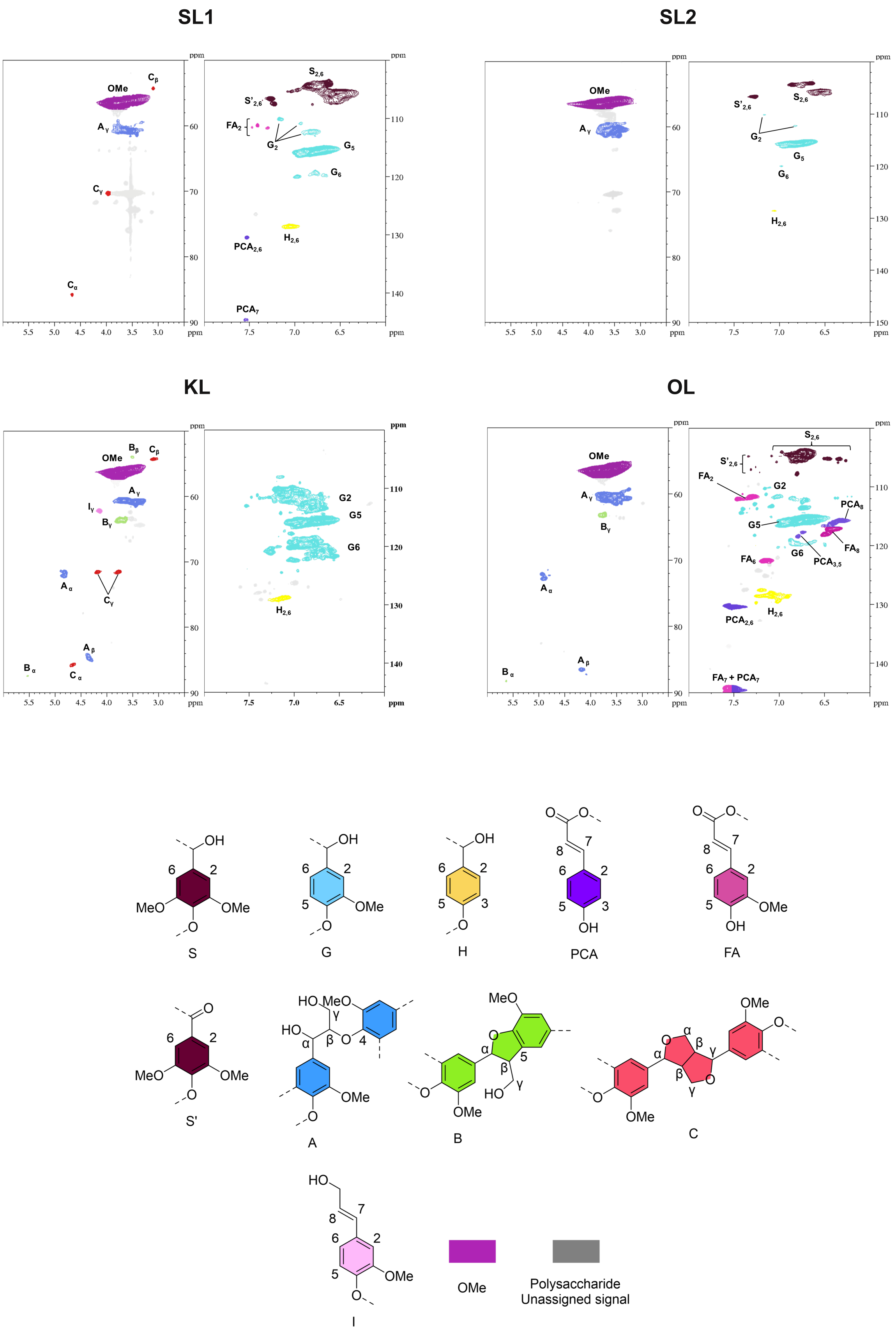

Figure S4. 2D HSQC NMR spectra of raw lignins.

DSC and TGA were employed to investigate thermal behavior and stability of the lignin samples. The second heating curves of the different samples, as displayed in Fig. S5a, give reliable estimates of the T_g. SL1 and SL2 exhibited T_g values of 99 °C and 128 °C respectively, while both KL and OL had higher T_g values of 146 °C and 157 °C, respectively. The observed trend in T_g values might be attributed to increases in molecular weight and increased chain stiffness resulting from a significant presence of aromatic rings in KL and OL compared with SL1 and SL2. TGA thermograms for various lignins analyzed are shown in Fig. S5c. The onset temperature ($T_{5\%}$) at which an initial weight loss event was observed due to moisture removal varied between 65 and 71 °C. As the temperature continued to increase, the onset of degradation was observed in the range of ~210-260 °C. The temperature of maximum degradation rate (T_{max}) associated with the fragmentation of the interunit linkages and resulting in the release of volatile products was observed at ~340–360 °C for all the samples. SL1 lost ~66% of its initial weight, while SL2, OL, and KL showed weight losses of ~64%, ~63%, and ~56%, respectively. This difference in weight loss may be explained by variations in molecular weight, as lignins with higher molecular weights typically exhibit increased char formation. This relationship is reflected in KL, which, possessing the highest molecular weight, demonstrated the lowest weight loss. Similarly, OL exhibited slightly lower weight loss compared to SL1 and SL2, consistent with its relatively higher molecular weight. Finally, in the case of SL1 and SL2, which have comparable molecular weights, the small difference observed may be due to hydroxyl group content, with the higher OH concentration in SL1 expected to facilitate dehydration reactions during thermal decomposition, resulting in a greater overall mass loss.

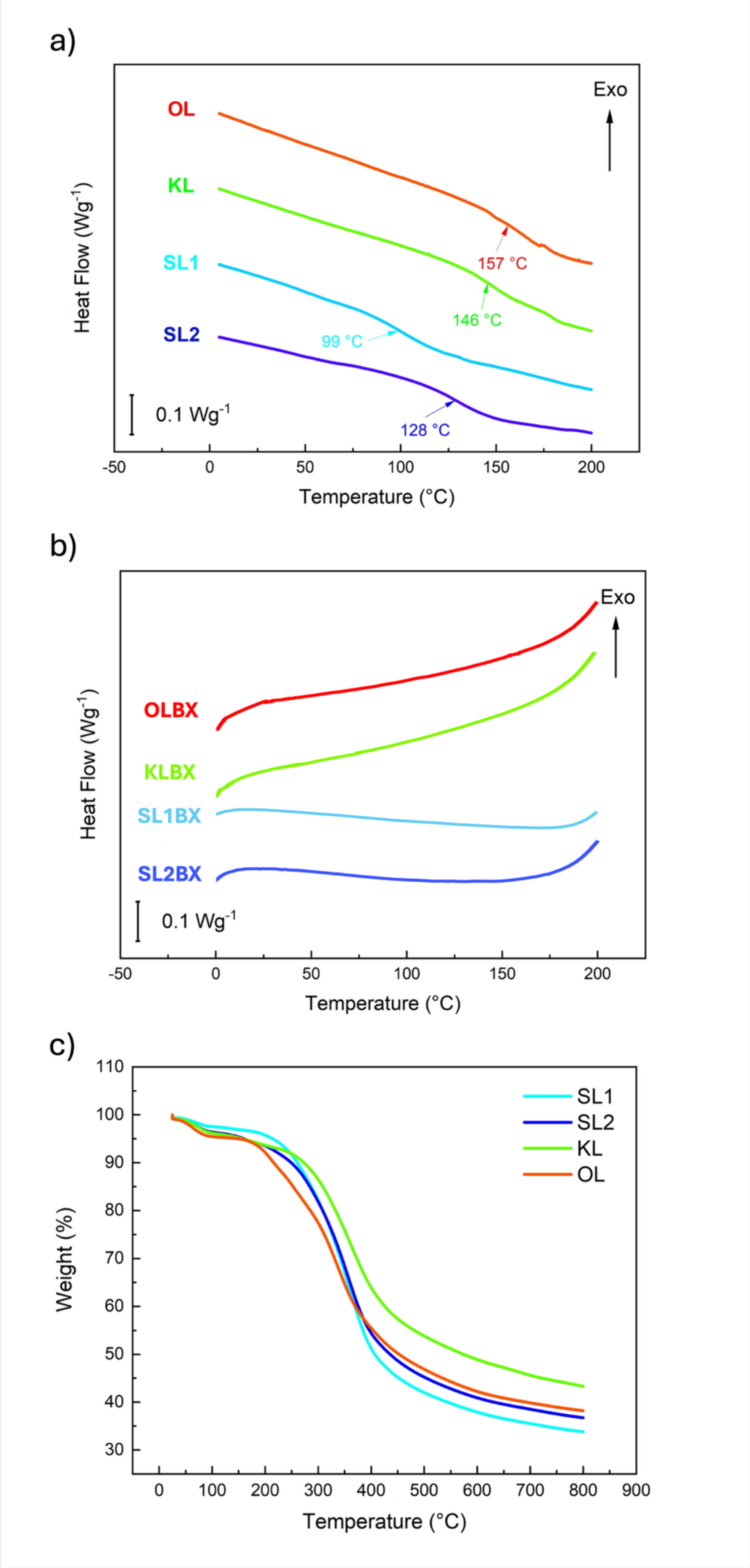

Figure S5. (a) DSC profiles of raw lignins (second heating cycle), (b) DSC profiles of the corresponding xerogels (second heating cycle), and (c) TGA curves of raw lignins (in nitrogen).

2. Lignin-Based Xerogels

Table S2. Screening of reaction parameters in the Lignin/5MF sol–gel system.

Lignin (g)		5MF (g)	Catalyst (g)		Solvent (mL)		Water (mL)	T (°C)	Time (h)	Result
SL1	1	0.66	H_2SO_4 8M	0.003	EtOH	-	1	90	6	NG
SL1	1	0.66	H_2SO_4 8M	0.03	EtOH	-	1	90	6	IG
SL1	1	0.66	H_2SO_4 8M	0.3	EtOH	-	1	90	6	IG
SL1	1	0.66	H_2SO_4 8M	1	EtOH	-	1	90	6	IG
SL1	1	1	H_2SO_4 8M	1	EtOH	-	1	90	6	IG
SL1	1	1	H_2SO_4 8M	1	EtOH	-	1	90	12	IG
SL1	1	1	H_2SO_4 8M	1	EtOH	1	1	90	6	IG
SL1	1	1	H_2SO_4 8M	1	EtOH	2	1	90	6	IG
SL1	1	1	H_2SO_4 8M	1	EtOH	1	2	90	6	IG
SL1	1	1	H_2SO_4 8M	1	EtOH	1	4	90	6	NG
SL1	1	5	H_2SO_4 8M	1	EtOH	4	1	90	6	IG
SL1	1	5	H_2SO_4 8M	1	EtOH	4	1	90	24	IG
**SL1**	**1**	**5**	**H_2SO_4 8M**	**1**	**EtOH**	**4**	**1**	**90**	**48**	**G**
SL1	1	5	*p*TSA	1	EtOH	4	1	90	48	IG
**SL1**	**1**	**5**	***p*TSA**	**2**	**EtOH**	**4**	**1**	**90**	**48**	**G**
SL1	1	5	HCl	2	EtOH	4	1	90	48	IG
SL1	1	5	H_3PO_4	2	EtOH	4	1	90	48	NG
SL1	1	5	Formic acid	2	EtOH	4	1	90	48	NG
SL1	1	5	Acetic acid	2	EtOH	4	1	90	48	NG
SL1	1	1	NaOH 1M	1	EtOH	4	-	90	6	NG
**SL1**	**1**	**5**	**H_2SO_4 8M**	**1**	**MeOH**	**4**	**1**	**90**	**48**	**G**
SL1	1	5	H_2SO_4 8M	1	*n*-BuOH	4	1	90	48	IG
SL1	1	5	H_2SO_4 8M	1	Cyclohexanol	4	1	90	48	IG
SL1	1	5	H_2SO_4 8M	1	DMSO	4	1	90	48	NG
SL1	1	5	H_2SO_4 8M	1	1,4-Dioxane	4	1	90	48	NG
**SL2**	**1**	**5**	**H_2SO_4 8M**	**1**	**EtOH**	**4**	**1**	**90**	**48**	**G**
**SL2**	**1**	**5**	***p*TSA**	**2**	**EtOH**	**4**	**1**	**90**	**48**	**G**
**KL**	**1**	**5**	**H_2SO_4 8M**	**1**	**EtOH**	**4**	**1**	**90**	**48**	**G**
**KL**	**1**	**5**	***p*TSA**	**2**	**EtOH**	**4**	**1**	**90**	**48**	**G**

**OL**	**1**	**5**	**H_2SO_4 8M**	**1**	**EtOH**	**4**	**1**	**90**	**48**	**G**
**OL**	**1**	**5**	***p*TSA**	**2**	**EtOH**	**4**	**1**	**90**	**48**	**G**

Notes: G: complete gelation; IG: incomplete gelation (partial network formation); NG: no gelation observed.

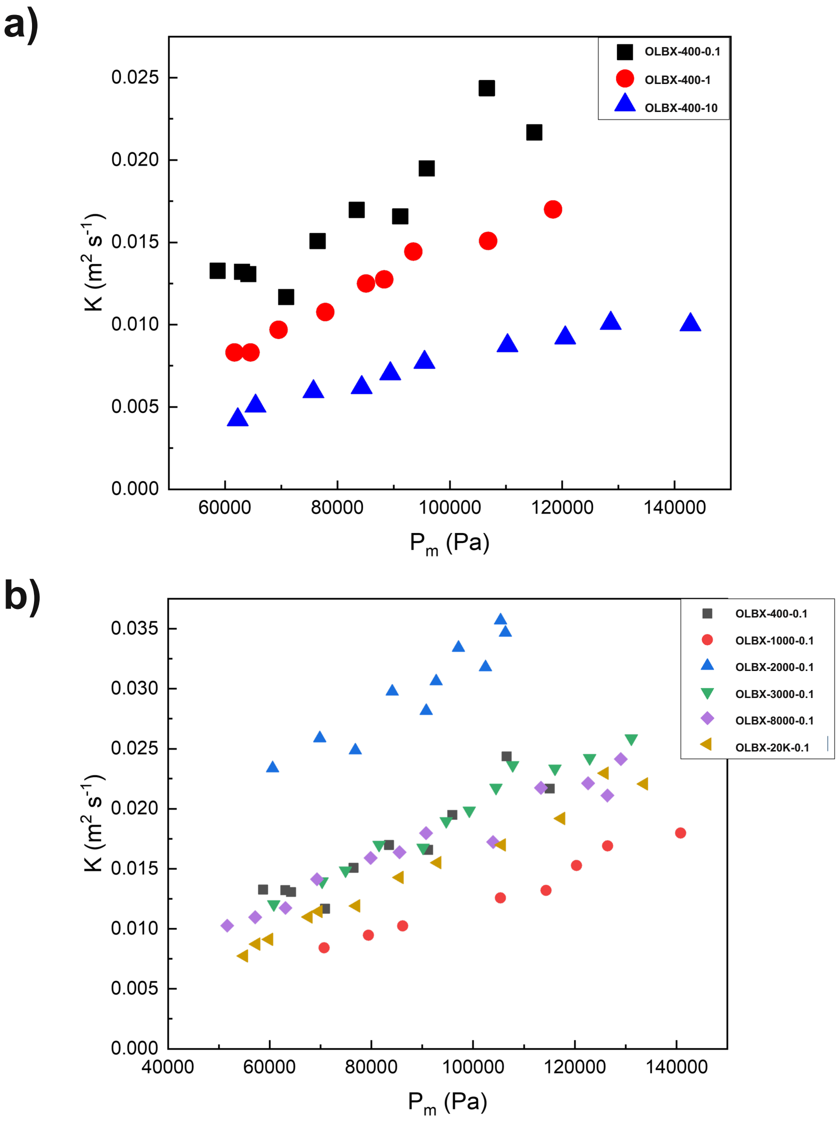

Figure S6. (a) Permeability coefficient *K* of lignin xerogels (produced using PEG having a M_w of 400 g/mol with a various PEG/OL ratios) as function of mean pressure P_m; (b) Permeability coefficient *K* of lignin xerogels (produced using PEG having various M_w with a PEG/OL ratio of 1:10) as function of mean pressure P_m.

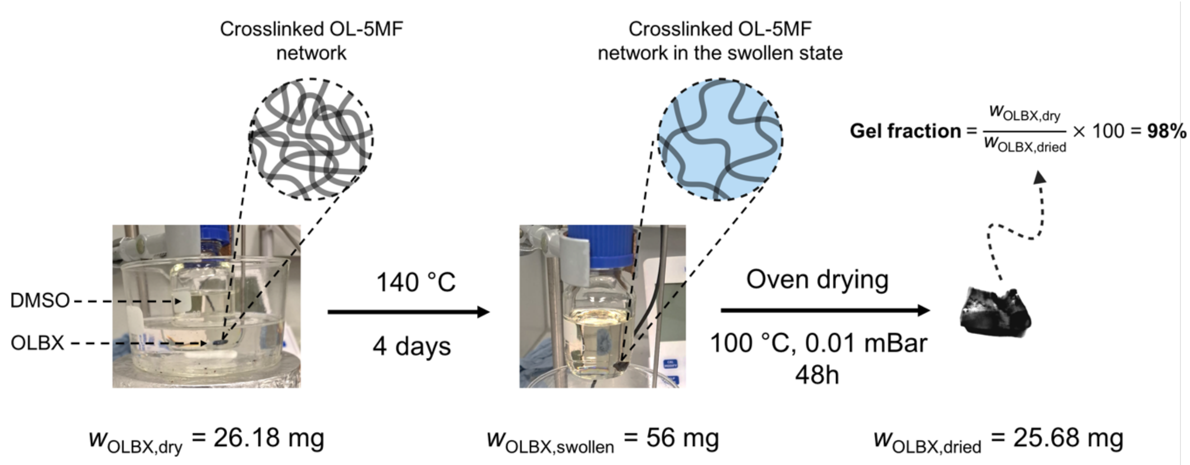

Figure S7. Schematic illustration of the gel fraction determination. A dry OLBX sample was swollen in DMSO and subsequently dried to remove the soluble components. The remaining insoluble residue corresponds to the gel fraction.

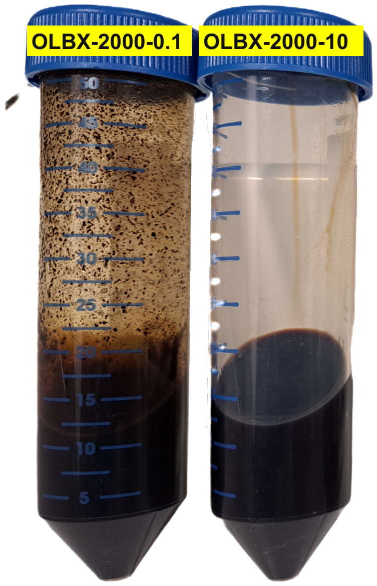

Figure S8. Digital images of OL / PEG / ethanol / water mixtures showing the improved solubility of OL as the PEG/OL ratio increases from 0.1:1 (left) to 10:1 (right).

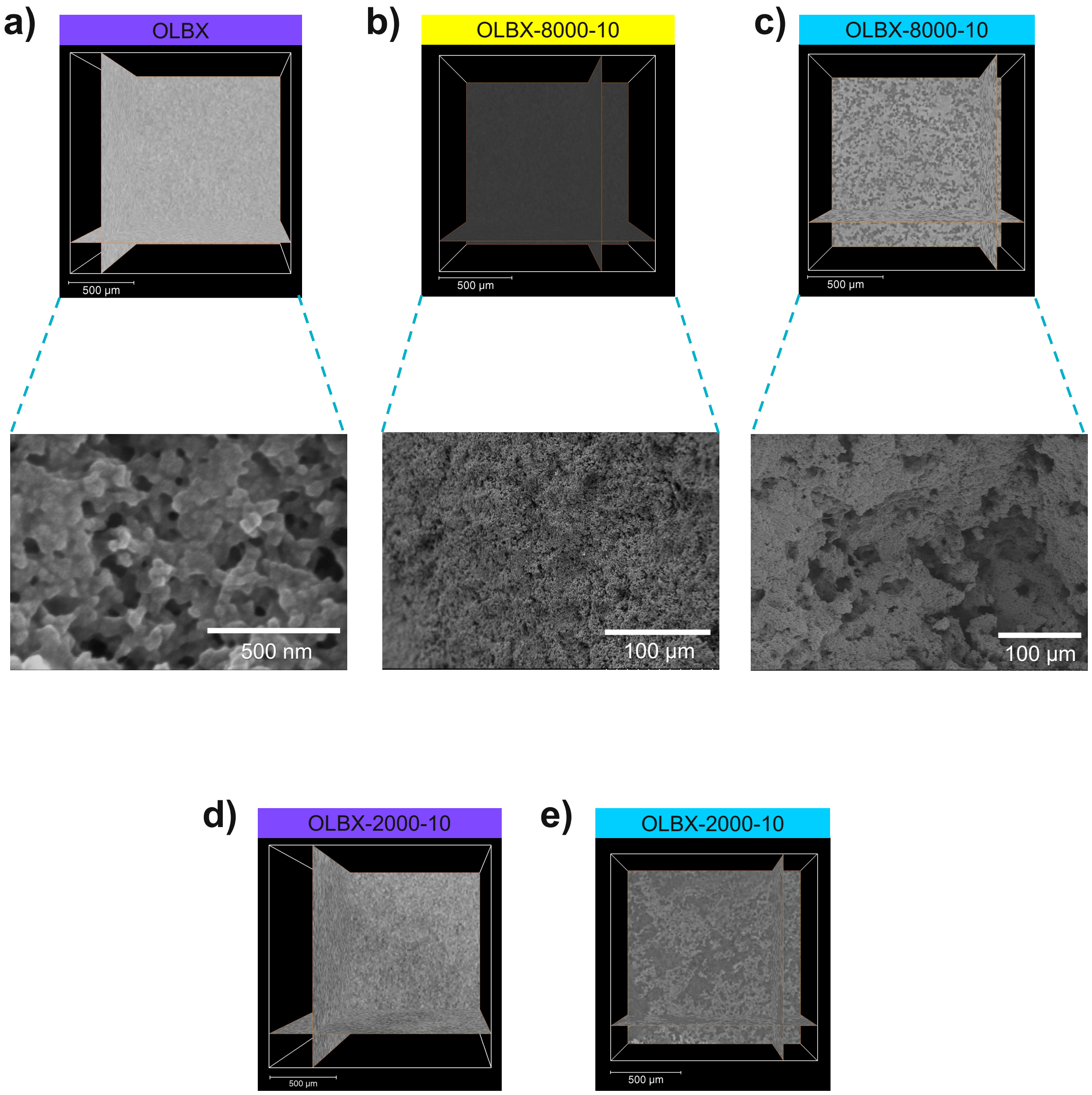

Figure S9. Orthogonal slices obtained by micro-CT of LBX samples prepared under varying solvent compositions: (a) OLBX (1 mL water, 4 mL ethanol), (b) OLBX-8000-10 (1 mL water, 8 mL ethanol), (c) OLBX-8000-10 (3 mL water, 4 mL ethanol) with corresponding SEM images, (d) OLBX-2000-10 (1 mL water, 4 mL ethanol), and (e) OLBX-2000-10 (3 mL water, 4 mL ethanol).

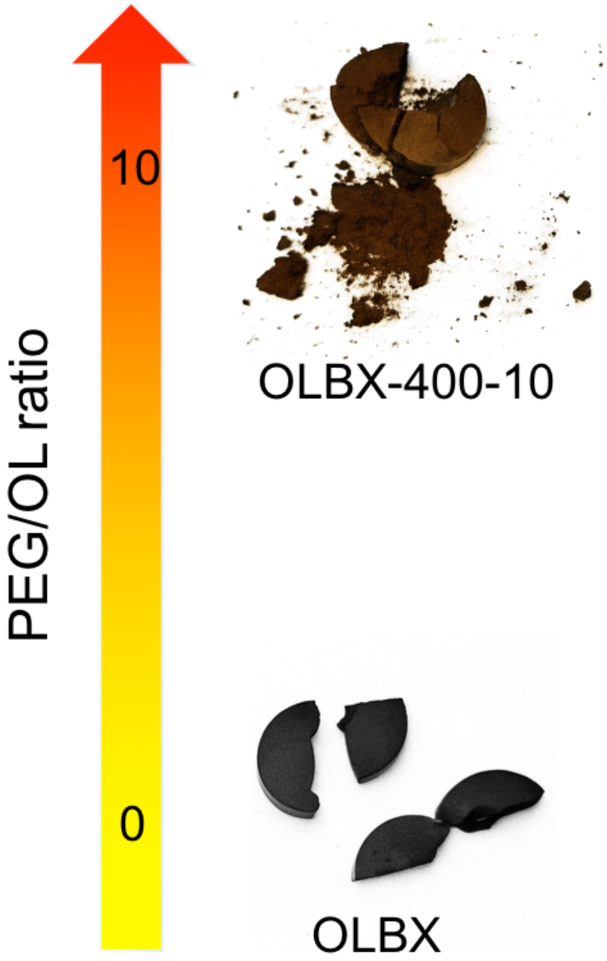

Figure S10. Fracture behavior of OLBX sample prepared without PEG (brittle, breaking into pieces) and OLBX-400-10 sample prepared in presence of 10 g of PEG (friable, fragmenting into powder), illustrating how PEG addition affect the mechanical properties of the LBX.

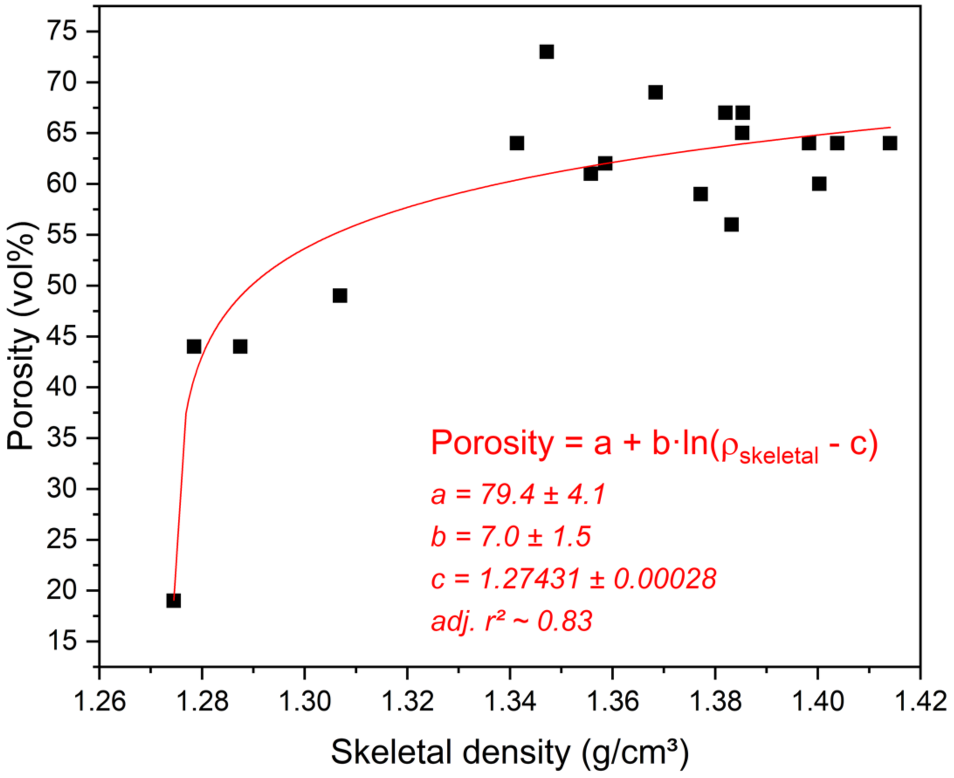

Figure S11. Correlation between OLBX porosity as measured via the envelope density method and skeletal density as measure via helium pycnometry, illustrating the relationship between low skeletal density and shrinkage during the xerogel preparation process (as indicated by reduced porosity).

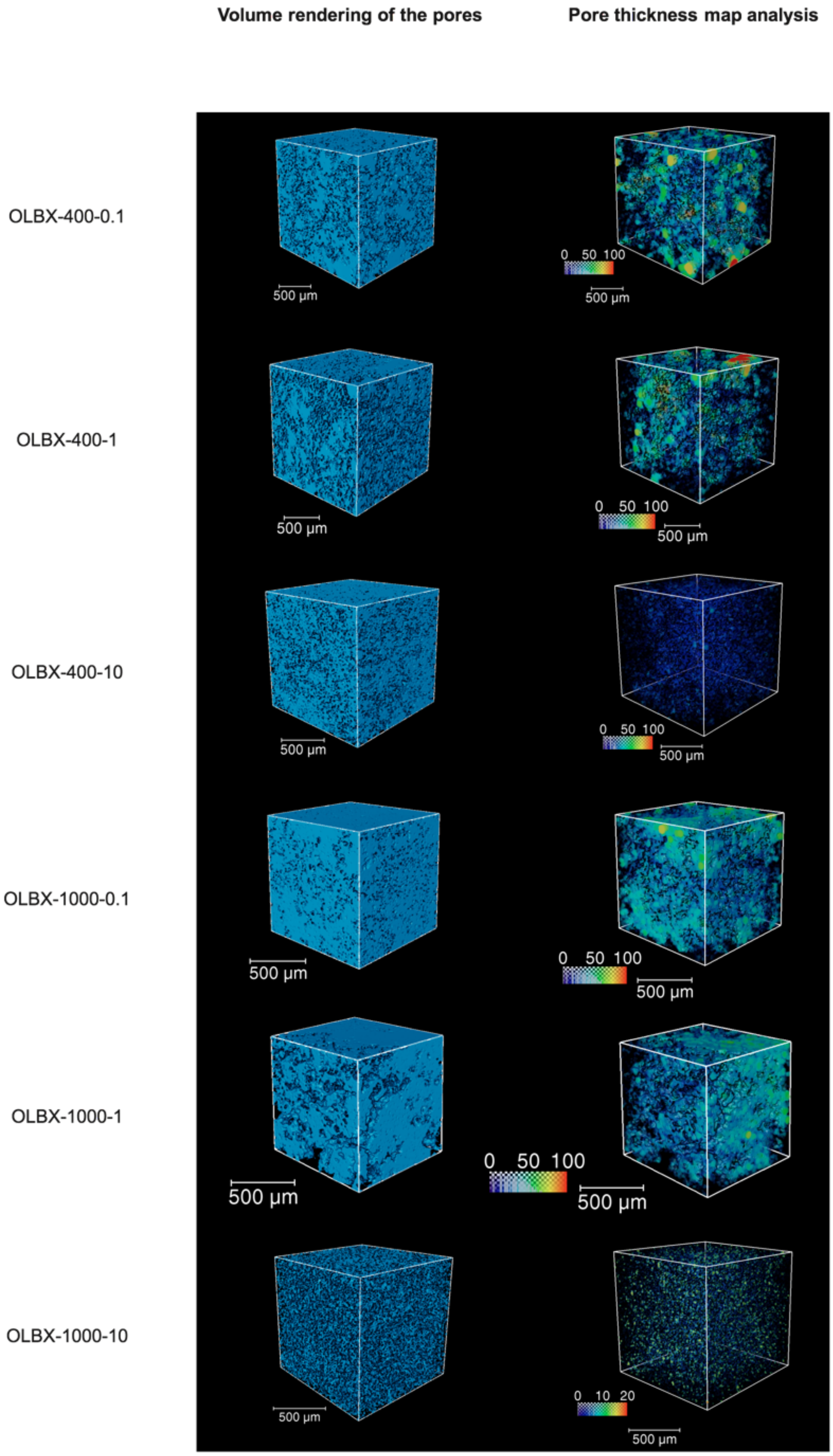
Volume rendering of the pores
Pore thickness map analysis
OLBX-400-0.1
OLBX-400-1
OLBX-400-10
OLBX-1000-0.1
OLBX-1000-1
OLBX-1000-10
500 µm
0 50 100
0 10 20

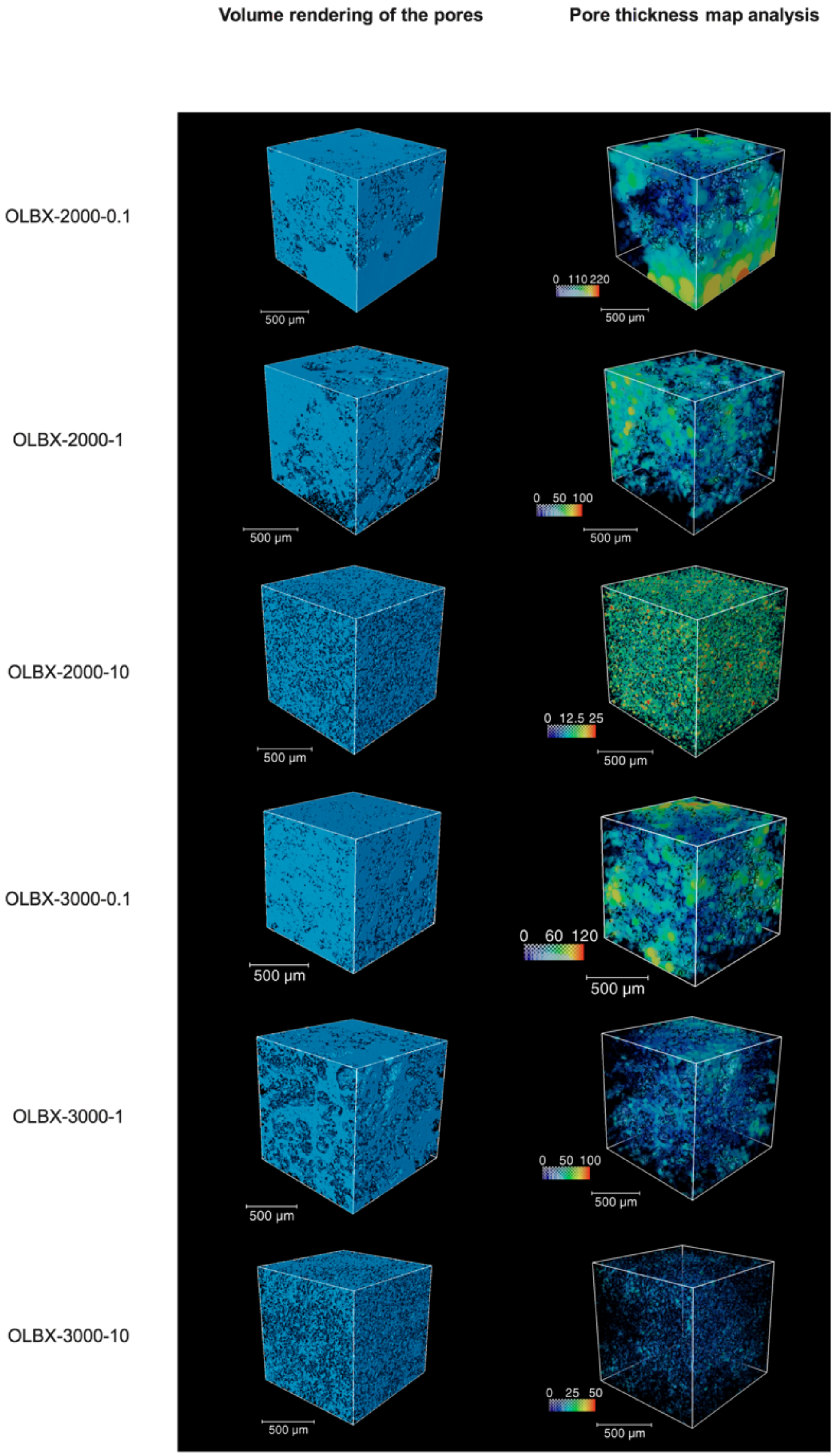

Volume rendering of the pores
Pore thickness map analysis
OLBX-2000-0.1
OLBX-2000-1
OLBX-2000-10
OLBX-3000-0.1
OLBX-3000-1
OLBX-3000-10
0 110 220
0 50 100
0 12.5 25
0 60 120
0 50 100
0 25 50
500 μm

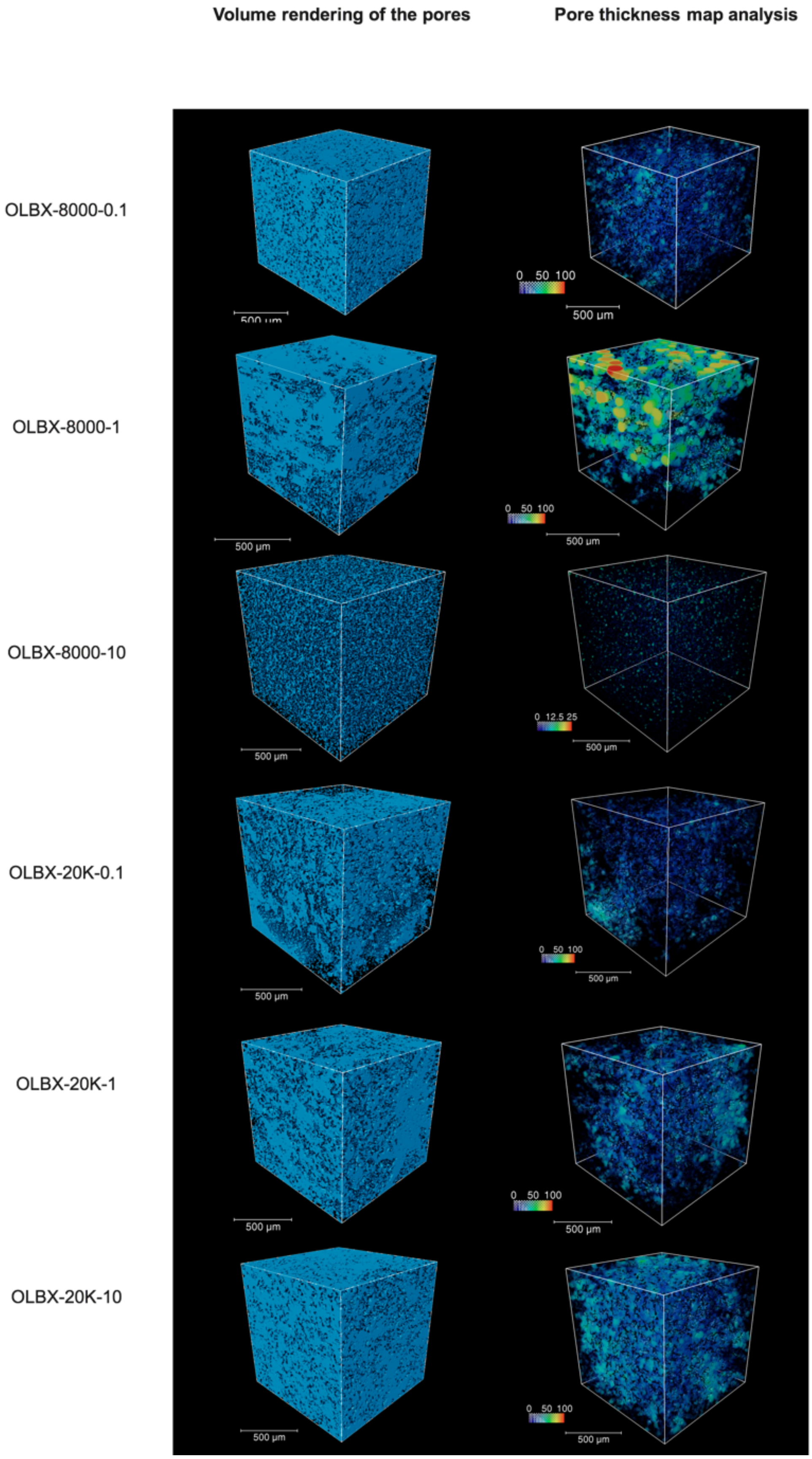

Figure S12. Volume rendering of the pores and pore thickness map analysis of LBX samples obtained by micro-CT. The color scale represents variations in the diameter (in µm) of the largest sphere fitting through a given pore.

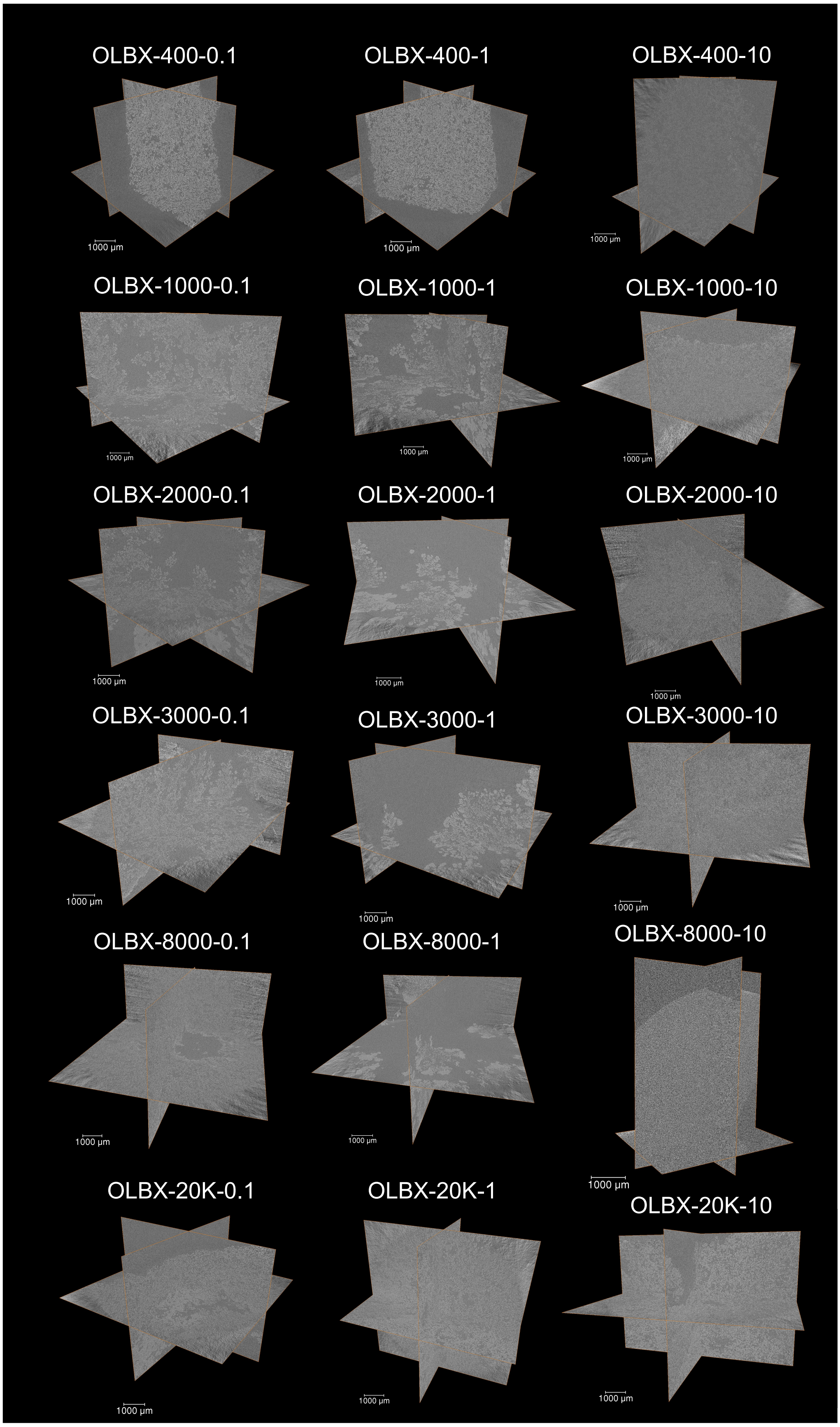

Figure S13. Large-scale orthogonal slices of LBX samples obtained by micro-CT.

References

(1) Wen, J.; Sun, S.; Xue, B.; Sun, R.-C. Recent advances in characterization of lignin polymer by solution-state nuclear magnetic resonance (NMR) methodology. *Materials* **2013** 6, 359–391.

(2) Jiang, X.; Savithri, D.; Du, X.; Pawar, S.; Jameel, H.; Chang, H.-m.; Zhou, X. Fractionation and characterization of kraft lignin by sequential precipitation with various organic solvents. *ACS Sustainable Chemistry & Engineering* **2017**, 5 (1), 835-842.

(3) Liu, L.-Y.; Patankar, S. C.; Chandra, R. P.; Sathitsuksanoh, N.; Saddler, J. N.; Renneckar, S. Valorization of bark using ethanol–water organosolv treatment: isolation and characterization of crude lignin. *ACS Sustainable Chemistry & Engineering* **2020**, 8 (12), 4745-4754.

(4) Tran, F.; Lancefield, C.; Kamer, P.; Lebl, T.; Westwood, N. Selective modification of the β–β linkage in DDQ-treated Kraft lignin analysed by 2D NMR spectroscopy. *Green Chemistry* **2015**, 17 (1), 244-249.
